# Erythroid Differentiation and Heme Biosynthesis Are Dependent on a Shift in the Balance of Mitochondrial Fusion and Fission Dynamics

**DOI:** 10.3389/fcell.2020.592035

**Published:** 2020-11-23

**Authors:** Alvaro M. Gonzalez-Ibanez, Lina M. Ruiz, Erik Jensen, Cesar A. Echeverria, Valentina Romero, Linsey Stiles, Orian S. Shirihai, Alvaro A. Elorza

**Affiliations:** ^1^Institute of Biomedical Sciences, Faculty of Medicine and Faculty of Life Sciences, Universidad Andres Bello, Santiago, Chile; ^2^Millennium Institute on Immunology and Immunotherapy, Santiago, Chile; ^3^Instituto de Ciencias Biomédicas, Universidad Autónoma de Chile, Santiago, Chile; ^4^Facultad de Medicina, Universidad de Atacama, Copiapó, Chile; ^5^Centro de Nanotecnología Aplicada, Facultad de Ciencias, Universidad Mayor, Santiago, Chile; ^6^Department of Medicine, Endocrinology, David Geffen School of Medicine, University of California, Los Angeles, Los Angeles, CA, United States; ^7^Metabolism Theme, David Geffen School of Medicine, University of California, Los Angeles, Los Angeles, CA, United States

**Keywords:** mitochondria, stem cell, permeability transition pore, erythropoiesis, fission and fusion, cell differentiation

## Abstract

Erythropoiesis is the most robust cellular differentiation and proliferation system, with a production of ∼2 × 10^11^ cells per day. In this fine-tuned process, the hematopoietic stem cells (HSCs) generate erythroid progenitors, which proliferate and mature into erythrocytes. During erythropoiesis, mitochondria are reprogrammed to drive the differentiation process before finally being eliminated by mitophagy. In erythropoiesis, mitochondrial dynamics (MtDy) are expected to be a key regulatory point that has not been described previously. We described that a specific MtDy pattern occurs in human erythropoiesis from EPO-induced human CD34^+^ cells, characterized predominantly by mitochondrial fusion at early stages followed by fission at late stages. The fusion protein MFN1 and the fission protein FIS1 are shown to play a key role in the progression of erythropoiesis. Fragmentation of the mitochondrial web by the overexpression of FIS1 (gain of fission) resulted in both the inhibition of hemoglobin biosynthesis and the arrest of erythroid differentiation, keeping cells in immature differentiation stages. These cells showed specific mitochondrial features as compared with control cells, such as an increase in round and large mitochondrial morphology, low mitochondrial membrane potential, a drop in the expression of the respiratory complexes II and IV and increased ROS. Interestingly, treatment with the mitochondrial permeability transition pore (mPTP) inhibitor, cyclosporin A, rescued mitochondrial morphology, hemoglobin biosynthesis and erythropoiesis. Studies presented in this work reveal MtDy as a hot spot in the control of erythroid differentiation, which might signal downstream for metabolic reprogramming through regulation of the mPTP.

## Highlights

- Impaired mitochondrial fusion prevents erythroid progression, heme biosynthesis and proper mitochondrial function, keeping cells mostly in progenitors and proerythroblast stage.
- Mitochondrial dynamics signaling for erythroid differentiation involves FIS1 and the mPTP.

## Introduction

Mitochondria play several critical roles throughout erythroid differentiation to produce red blood cells. In early expansion of erythropoiesis -from hematopoietic stem cell (HSC) to CFU- E-, mitochondria are involved in stemness and stem cell differentiation, regulating energy metabolism and metabolic reprogramming from glycolytic and proliferative, to oxidative and differentiating metabolism (Ahlqvist et al., 2015; Bonora et al., 2015; Schell and Rutter, 2017). In late expansion -from proerythroblasts to polychromatophilic erythroblasts-, mitochondria are involved in iron metabolism and heme biosynthesis (Fontenay et al., 2006; Dailey and Meissner, 2013). Terminal erythroid maturation, from orthochromatic erythroblasts to mature erythrocytes, is distinguished by mitochondria driving their own elimination through mitophagy to produce a fully mature erythrocyte (Moras et al., 2017).

Mitochondrial dynamics (MtDy) refers to continuous fission and fusion events that participate in mitochondrial turnover, coupled to mitophagy, and cell signaling. These interactions, in terms of frequency and remodeling of the mitochondrial web, reflect mitochondrial adaptive responses to accomplish cell proliferation, differentiation, energy demand, stress response, cell survival, and death (Westermann, 2010; Wilson et al., 2013; Shaughnessy et al., 2014). Mitochondrial fission is dependent on the protein DRP1 that has been recognized as a mitochondrial fission promoter (Youle and van der Bliek, 2012; Held and Houtkooper, 2015). It is located in the cytosol, but translocates to mitochondria to bind the mitochondrial receptor proteins FIS1, MFF, MiD49, and MiD51 (Gandre-Babbe and van der Bliek, 2008; Losón et al., 2013), which are all located in the mitochondrial outer membrane (MOM). Mitochondrial fusion is dependent on MFN1 and MFN2, also located in the MOM; and OPA1, which is located in the mitochondrial inner membrane (Kluge et al., 2013; Liesa and Shirihai, 2013).

Mitochondria in stem cells feature low biomass and mtDNA copy number and immature cristae (Wanet et al., 2015). OXPHOS capacity and ROS generation are minimal, and the mitochondrial membrane potential is mainly sustained by the ATP synthase in reverse mode i.e., consuming glycolysis-made ATP for protons translocation. Upon differentiation, mitochondria undergo fusion, increasing their respiratory capacity, mitochondrial membrane potential, and ROS generation; switching from glycolytic to oxidative metabolism (Zhang et al., 2011; Maryanovich and Gross, 2013; Bigarella et al., 2014; Teslaa and Teitell, 2015; Wanet et al., 2015; Prieto and Torres, 2017). In this regard, MtDy act as a modulator of stemness and stem cell fate. It has been shown that deregulation of the mitochondrial fission protein DRP1 was associated with a loss of pluripotent markers (Nanog, Oct4, and Ssea) in mouse embryonic stem cells (Todd et al., 2010). Furthermore, cell differentiation from murine mesenchymal stem cells into osteo, chondro, and adipocytes requires specific MtDy changes. In adipo and osteogenesis, mitochondrial elongation and expression of MFN1 and MFN2 are increased in contrast to chondrogenesis, where mitochondrial fragmented morphology and expression of DRP1 and FIS1 are favored (Forni et al., 2016). The right balance of the mitochondrial fusion/fission ratio has also been described in the immune system by controlling macrophage migration (Campello et al., 2006); antiviral signaling (Pourcelot and Arnoult, 2014), and T-cell fate through metabolic reprogramming (Buck et al., 2016).

Erythropoiesis is the only system in nature capable of producing more than 200 billion cells per day starting from stem cells. This system involves exceptional high rates of proliferation and differentiation where the role of MtDy has not been studied. Our group has previously shown that exposure to non-cytotoxic copper overload or deficiency in erythropoietic cells modifies cell proliferation and differentiation along with changes in mitochondrial morphology, expression of MtDy proteins, and the rate of mitochondrial fission and fusion events (Ruiz et al., 2016; Jensen et al., 2019). In this work, we hypothesized that a specific pattern of MtDy is needed for the appropriate commitment and differentiation of HSC into the red cell lineage.

## Materials and Methods

### Cell Isolation, Culture and Lentiviral Transduction

Mouse G1E-ER cells a murine erythroleukemia cell line that synchronously undergoes differentiation when exposed to β-estradiol, were cultured in Iscove’s Modified Dulbecco’s medium (Invitrogen) supplemented with 15% heat-inactivated fetal bovine serum, 2% pen/strep (Invitrogen), 120 nM monothioglycerol (Sigma), 2 U/ml EPO (StemCell Tech), and 50 ng/ml SCF/KL (StemCell Tech). Erythroid differentiation was induced by adding 10^–6^ M β-estradiol to cells.

Primary human CD34+ cells were obtained from umbilical cord blood (UCB), after a normal full-term delivery (informed consent was given). Briefly, UCB was collected in Terumo bags (PB-1PD256Z9s) containing CPD anticoagulant. Next, UCB was diluted 1:1 in D-PBS, layered on top of Lymphocyte Separation Medium (25-072-CV, Corning) in 2:1 ratio in a 50 ml conical centrifuge tube and centrifuged at 900 × *g* for 30 min, room temperature (RT), brake-off. The mononuclear layer was collected and washed three times with D-PBS/1% FBS to proceed with CD34+ cell isolation by immune-magnetic separation using the MiniMacs CD34 MicroBead Kit, human (Miltenyi Biotec) according to the manufacturer instructions. Isolated CD34+ cells were cultured in 24-well tissue culture plates containing 0.5 mL culture medium/well in a density of 2 × 10^5^ cells per well. The culture medium contained 10 μg/mL recombinant human insulin (I9278-5 mL, Sigma), 120 μg/mL human holotransferrin (41-952-100, Biological Industries), 1× Glutamax (35050-061, Gibco), 30% FBS, 1× Penicillin-Streptomycin, 10 ng/mL Human IL-3 (130-093-908, MACS), 100 ng/mL Human SCF (130-096-692, MACS), 3 U/mL recombinant human EPO (287-TC, RD Systems) in Iscove’s modified Dulbecco’s medium (01-058-1, Biological industries) (Giarratana et al., 2005). Cultures were incubated at 37°C in 5% CO_2_.

Lentiviral particles were produced in HEK-293T cells which were co-transfected with the plasmids psPAX2 (12259, Addgene), pMD2.G (12259, Addgene), and either pLVCTH or pWPI. pLVCTH-shFIS1 and pLVCTH-shMFN1 were used for gene silencing and pWPI FIS1 and pWPI MFN1, for gene overexpression. Lentiviral particles were added to 5 × 10^4^ cells with 8 μg/mL Polybrene (107689, Sigma) and exposed overnight in a 96-well plate.

### Erythroid Progression

Erythroid cells were immunostained with phycoerythrin (PE)–conjugated anti-human CD235a (1:100) (116207, BioLegend) and Allophycocyanin (APC)–conjugated anti-human CD71 (1:100) (551374, BD Pharmingen) antibodies. Flow cytometry was carried out on a BD Accuri C6 Cytometer (BD Biosciences). In addition, 3,3,5,5-tetramethylbenzidine (Sigma-Aldrich) was used for benzidine staining according to Osti et al. (1997).

### Real-Time qPCR

Total RNA was isolated with the PureLink RNA Mini Kit (12183018A, Thermo) and cDNA was synthesized with ProtoScript First Strand cDNA (E6300S, New England Biolabs). RT-PCR was performed with the Power SYBR Green PCR Master Mix 2× (4367659, Thermo). Primers are listed in Supplementary Table S1.

### Immunoblotting

Cell pellets were lysed in RIPA buffer containing 1× Halt^TM^ protease inhibitor cocktail (78437, Thermo) and supernatants collected after 10 min centrifugation at 12000 × *g* for SDS-PAGE. Total proteins were transferred to PVDF membranes and blotted with the antibodies against FIS1 (ab71498, Abcam), MFN1 (ab104274, Abcam), β-Actin (ab8227, Abcam), Caspase-3 (9665, Cell Signaling), Caspase-8 (sc-5263, Santa Cruz), Caspase-9 (sc-8355, Santa Cruz), HSP70 (TA309356, Origene) and Total OXPHOS Human WB Antibody Cocktail (ab110411, Abcam).

### Mitochondrial Membrane Potential and ROS Measurements

Changes in the mitochondrial membrane potential (mtΔΨ) were determined using the potentiometric fluorescent probe tetramethylrhodamine-ethyl-ester (TMRE) (Life Technologies) in non-quenching mode. For confocal microscopy visualization, cells were adhered to Poly-L-lysine (P4707, Sigma) - coated coverslips and placed in a temperature-controlled and CO_2_-supplemented chamber (Chamlide TM IC, Live Cell Instrument Inc.). Mitochondria were stained with 10 nM TMRE for 20 min at 37°C. Z-stack images were acquired with a slice separation of 1 μm throughout the entire cell depth with Leica TCS LSI confocal microscope and further analyzed with the Image J software (Molecular Devices). For Flow Cytometry analysis, cells were resuspended in FACS buffer (PBS/1% FBS) and mitochondria stained with 10 nM TMRE for 20 min at 37°C. As a negative control, TMRE-stained cells were treated with 5 μM FCCP (carbonyl cyanide-p-trifluoromethoxyphenylhydrazone) (C2920 Sigma-Aldrich) for 10 min to depolarize mitochondria. Flow cytometry was carried out on a BD Accuri C6 Cytometer (BD Biosciences). Changes in mitochondrial ROS were determined using MitoSOX^TM^ Red Mitochondrial Superoxide Indicator (M36008, Thermo Fisher). Cells were resuspended in FACS buffer, loaded with 5 μM of MitoSOX^TM^ for 10 min at 37°C and analyzed immediately by Flow Cytometry. MitoSOX results are displayed as the ratio MitoSOX/TMRE because MitoSOX uptake by mitochondria is dependent on membrane potential.

### Mitochondrial Morphology Analysis

Mitochondria were labeled by immunofluorescence or direct staining with MitoTracker Red CMXRos (M7512, Thermo Fisher). For the former, cells were attached to Poly-L-lysine (P4707, Sigma) coated slides, fixed with 4% paraformaldehyde (15711, Electron Microscopy Sciences) for 10 min at RT and then permeabilized with 0,1% Triton X-100 at RT for 5 min. After washing with PBS for two times, cells were incubated in blocking buffer PBS/1% BSA for 30 min at RT followed by overnight incubation with the Anti-VDAC1 primary antibody (1:1000) (ab15895, Abcam) at 4°C. After 2 washes in PBS, cells were incubated with the Alexa Fluor 594 secondary antibody (1:1000) (A11037, Life) for 2 h. at RT. Cells were mounted with DAPI Fluoromount-G (0100-20, Southern Biotech) and examined with a Leica TCS LSI confocal microscope. In direct staining, cells were stained with 10 nM MitoTracker Red CMXRos for 20 min at 37°C, washed twice in D-PBS, fixed in 4% paraformaldehyde (15711, Electron Microscopy Sciences), and mounted with DAPI Fluoromount-G (0100-20, Southern Biotech).

Z-stacks images were acquired with a slice separation of 1 μm throughout the entire cell depth and analyzed with the FIJI-ImageJ software (Schneider et al., 2012). Image processing consisted of background subtraction, threshold set, and analyze particle to obtain area, perimeter, and circularity descriptors. Each Z-stack obtained approximately 400 mitochondrial units. Data were plotted as frequency histograms for circularity and scatter plots Area/Circularity or Perimeter/Circularity. Scatter plots with histograms were done with the Python-based software Matplotlib 3.3.0.

### Transmission Electron Microscopy

For ultrastructure analyses of mitochondria, cells were fixed in 2.5% glutaraldehyde and 0.1 M cacodylate buffer, pH 7.2, for 6 h at RT, and then rinsed with a 0.1 M cacodylate buffer, pH 7.2, for 18 h at 4°C. Right after, cells were immersed in 1% OsO_4_ for 90 min, rinsed with distilled water, and labeled with 1% uranyl acetate for 1 h. Cells were dehydrated in graded series of acetone to 100% and embedded in Epon resin. Polymerization reactions were done at 60°C for 24 h. Ultrathin sections (60–70 nm) were obtained with the ultramicrotome Sorvall MT-500, disposed in copper grids, and contrasted with 4% uranyl acetate in methanol for 2 min and lead citrate for 5 min. Grids were examined with a Philips Tecnai 12 electron microscope at 80 kV. Mitochondria from each transmission electron microscopy (TEM) image was hand-drawn on sulfuric acid translucent tracing paper which were scanned for ImageJ processing as described above.

### Statistical Analysis

Statistical analysis was done with GraphPad Prism 7 (GraphPad Software Inc) with a significance level α ≤ 0.05. ANOVA and Bonferroni *post hoc* tests were performed to compare averages values between groups. Frequency distribution of mitochondrial circularity data obtained from confocal and TEM images was analyzed through contingency tables and a Chi-square test. Area/Circularity and Perimeter/Circularity scatter plots were analyzed by linear regression. R^2^ and the Mean Square Error (MSE) (the lower the MSE the higher the accuracy of prediction) were also calculated.

## Results

### Mitochondrial Dynamics and Morphology in Erythropoiesis

Mitochondrial dynamics and morphology were first investigated in the mouse erythropoietic cell line G1E-ER (G1E cells expressing a GATA-1 construct fused to an estrogen receptor ligand-binding domain), given their fast and synchronic erythroid differentiation when stimulated with β-estradiol. Mitochondrial morphology and the expression of MtDy genes during erythroid differentiation were examined by confocal microscopy and quantitative PCR, respectively. 24 h after β-estradiol-induced erythropoiesis, the mitochondrial web changed from elongated to fragmented (Supplementary Figure S1A). The mRNA expression pattern of *fis1*, *opa1*, *mfn1*, and *mfn2* genes was measured over 48 h after β-estradiol-induced erythropoiesis at regular 12 h intervals by quantitative real-time PCR. The mRNA concentration of *fis1*, which ranged from 2.27 to 5.82 fM, was considerably greater than those of the *opa1*, *mfn1*, and *mfn2* fusion genes which were all under 1 fM at every time point. It was also observed that *mfn1* transcripts were tenfold more abundant than *mfn2* ones (Supplementary Figure S1B). While the gene expression of *opa1* and *mfn2* do not seem to change over mouse erythroid differentiation, *fis1* and *mfn1* genes showed a specific and differential mRNA expression pattern, progressively increasing their expression until 36 h followed by a decline at 48 h post β-estradiol treatment (Supplementary Figure S1B). These results suggest *fis1* and *mfn1* genes are playing a critical role in erythropoiesis and, therefore, were studied further.

**FIGURE 1:**
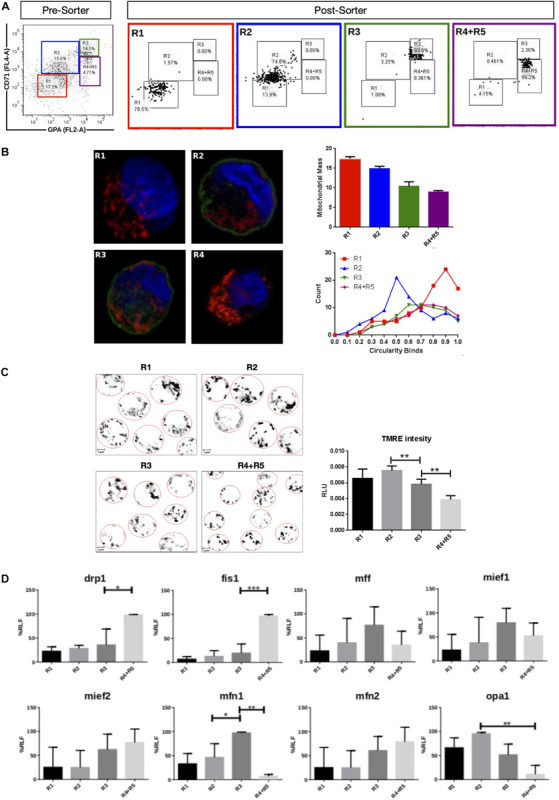
Mitochondrial features throughout erythroid progression. Human hematopoietic CD34+ stem cells isolated from umbilical cord were cultured and EPO-induced to erythropoiesis for 3, 5, 8, 10, 13, and 16 days and pooled together in just one pre-sort sample. **(A)** Cell separation of different erythroid populations. Pre-sort samples were labeled with CD71 and GPA surface markers to follow up erythroid progression. Five erythroid populations are distinguished: R1 progenitors, R2 proerythroblast, R3 basophilic erythroblast (these cells start making hemoglobin), R4 polychromatophilic erythroblast and R5 orthochromatophilic erythroblast and reticulocytes. After labeling, samples were sorted to obtain four highly pure cell populations as seen as in the post-sort panel for R1, R2, R3, and R4 + R5. **(B)** Mitochondrial biomass and morphology analysis. Each sorted cell population was stained with DAPI (nucleus, blue) and MitoTracker Red FM (mitochondria, red), and immunolabeled with anti CD71 antibody, green. Confocal microcopy was done to quantify mitochondrial mass and circularity, a form factor where 1 is a perfect circle and 0, an elongated or branched mitochondrion. From R1 to R4 + R5, mitochondrial biomass decreases, and circularity goes from round mitochondria in R1 to elongated in R2 and then to come back to intermediate values in R3 and R4 + R5. Mitochondrial biomass was calculated as the percentage of total cell volume. Statistical analysis was ANOVA followed by Bonferroni *post hoc* test. Mitochondrial Circularity was plotted as a frequency distribution and statistically analyzed through contingency tables and a Chi-square test (*p* < 0.05). **(C)** Mitochondrial membrane potential analysis. Sorted cell populations were stained with TMRE dye in a non-quenching mode and visualized under confocal microscope. Membrane potential goes from average in R1 to high in R2. Then, it starts to decrease toward R4 + R5 population. **(D)** Mitochondrial Dynamics gene expression analysis. Total RNA, cDNA synthesis and RT-PCR were performed in each sorted population for the mitochondrial fission genes *fis1*, *mff*, *mief1*, and *mief2*; and for the mitochondrial fusion genes *mfn1*, *mfn2*, and *opa1*. *18s* was used as a loading control. In general, fusion is predominant at early stages and the fission, at later stages of erythroid differentiation. All plotted values in panels **(C,D)** are ±SEMs of *n* = 3. Statistical analysis was ANOVA followed by Bonferroni *post hoc* test (^∗^ *p* < 0.05, ^∗∗^ *p* < 0.01, ^∗∗∗^ *p* < 0.001).

Mitochondrial dynamics and morphology in human erythropoiesis (Figure 1) were studied through *in vitro* differentiation of EPO-induced primary CD34+ HSCs that were isolated from UCB and cultured for up to 16 days. Samples were collected at days 0, 3, 5, 8, 10, 13, and 16 of differentiation, pooled, immunolabeled with anti-CD71-APC and anti-GPA-PE, and cell sorted by for FACS. Four cell populations, representing erythroid progression, were sorted out. R1, progenitors; R2, proerythroblasts; R3, basophilic erythroblasts (hemoglobin is synthesized); and R4 + R5, poly, and orthochromatophilic erythroblasts (Zhang, 2003; Figure 1A). Then, mitochondria of each cell population were stained with MitoTracker Red for mitochondrial biomass and circularity analysis (Figure 1B) or with TMRE for mitochondrial membrane potential analysis (Figure 1C). Cells were visualized by confocal microscope. Throughout erythroid differentiation, mitochondrial biomass continuously decreased from R1 (progenitors) to R4 + R5 (poly and orthochromatophilic erythroblasts). Circularity, a form factor where 1 is a perfect round mitochondrion and 0 a is filamented or branched mitochondrion, shows that the R1 population displayed round mitochondria with a circularity peak value of 0.9. R2 displayed a value of 0.5, meaning that mitochondria became more elongated. Mitochondria circularity moved back to 0.7 in R3 and R4 + R5, becoming again fragmented. Mitochondrial membrane potential (Figure 1C) increased from R1 to R2, and then decreased from R2 to R4 + R5. The more elongated mitochondria observed in R2 are concomitant with the highest membrane potential.

To explore MtDy gene expression, total RNA isolation and cDNA synthesis was performed for R1, R2, R3, and R4 + R5 erythroid populations. The expression analysis of the fission genes *fis1*, *drp1*, *mff*, *mief 1* and *mief 2*; and the fusion genes *mfn1*, *mfn2*, and *opa1* was assessed by relative RT-PCR (Figure 1D). For mitochondrial fission, significant differences were found for *fis1* and *drp1* transcripts, which were upregulated throughout differentiation reaching maximal expression in R4 + R5. *Fis1* and *Drp1* had 4 and 1.5 times more transcripts in R4 + R5 as compared with R3 population (*p* < 0.05). On the other hand, for mitochondrial fusion, a significant difference was found for *mfn1* which was upregulated in R3 having twice the transcripts than the R2 population. Non-significant differences were observed for *mff*, *mief1*, *mief2*, *mfn2*, and *opa1*. These results are in accordance with those obtained in mouse G1E-ER cells and suggest that *fis1* and *mfn1* are important genes for erythroid differentiation. Furthermore, the MtDy gene expression profile correlates with circularity values suggesting a predominant mitochondrial fusion from R1 to R2 where mitochondria go from fragmented to elongated; and then, predominant mitochondrial fission from R2 to R4 + R5 where mitochondria became fragmented again. Fragmented mitochondria with low membrane potential in the R4 + R5 population is imperative for mitochondrial clearance by mitophagy which begins in the orthochromatophilic erythroblasts (Betin et al., 2013).

### Functional Characterization of FIS1

**FIGURE 2:**
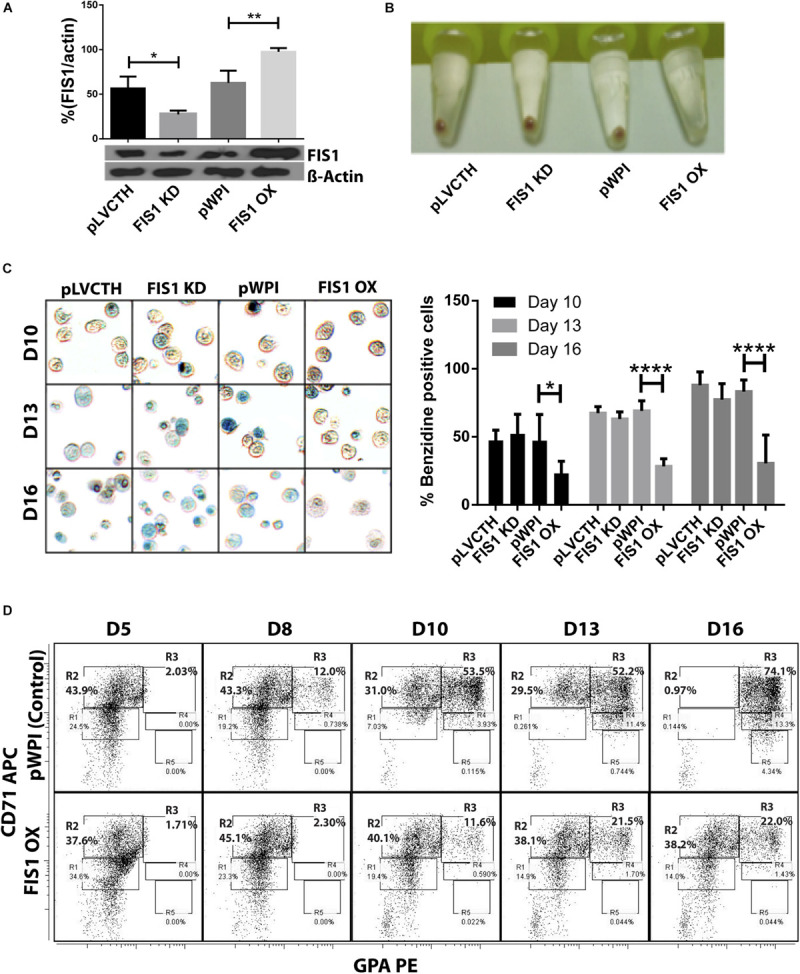
Functional characterization of FIS1 in erythropoiesis. **(A)** Western blot to detect the knock-down or over-expression of FIS1 protein. Quantification was performed by band densitometry. Values are ±SEM, *n* = 3. **(B)** Visual inspection of cellular pellet. FIS1 OX cells do not differentiate under EPO-induced erythroid differentiation. Cell cultures at D16 were spun down and the pellet’s color, visualized. Red pellets were observed in all conditions, but not in FIS1 OX cells whose pellet was white. **(C)** Quantification of hemoglobin carrying cells. Cells from D10, D13, and D16 of differentiation were staining with benzidine to detect hemoglobin. Blue cells (benzidine+) were counted. FIS1 OX cells do not make hemoglobin. Plotted values are ±SEM, *n* = 3. **(D)** Analysis of erythroid progression. FIS OX cells are mainly arrested at the level of progenitors and proerythroblasts. This was seen by flow cytometry analysis of control and FIS OX cells labeled with the surface markers anti-CD71-APC and anti-GPA-PE at D5, D8, D10, D12, and D16 of EPO-induced differentiation (*n* = 3). Statistical analysis, ANOVA followed by Bonferroni *post hoc* test (^∗^ *p* < 0.05, ^∗∗^ *p* < 0.01, ^****^ *p* < 0.001).

Loss and gain of function experiments for FIS1 were performed by transducing CD34+ cells with lentiviral particles containing either pLVCTH-siRNA-FIS1 construct to knock-down FIS1 (FIS1 KD), or pWPI-FIS1 (full cDNA) for FIS1 over-expression (FIS1 OX). Over 90% efficiency was obtained at D3 for both constructs as seen as by GFP positive expression with almost no dead cells (Supplementary Figure S2). FIS1 KD and FIS1 OX protein expression were confirmed by Western blot which showed a significant 66% reduction and 56% increase, respectively in the FIS1 protein levels (*p* < 0.01) as compared with controls (Figure 2A).

Transduced CD34+ cells were EPO-induced to erythroid differentiation for 16 days and spun down to observe their ability to differentiate into red blood cells by looking at the color of the pellet, which is expected to be red due to hemoglobin biosynthesis (Figure 2B). While the controls, pLVCTH and pWPI, and the FIS1 KD cells had an intense red pellet, the FIS1 OX cells showed a white cell pellet, suggesting a defect in erythropoiesis. To follow-up with hemoglobin biosynthesis, cells were collected at D10, D13, and D16, and benzidine-stained for hemoglobin detection (Figure 2C). Benzidine-positive cells (blue color cells) increased in pLVCTH, pWPI and FIS1 KD conditions reaching over 75% at D16. On the other hand, FIS1 OX cells barely reached 21% (*p* < 0.01).

To understand what stage of erythroid differentiation was affected by FIS1 OX, cells were collected at D5, D8, D10, D13, and D16, and immunophenotyped with the surface markers CD71 and GPA (Figure 2D) for flow cytometry analysis. FIS1 OX cells underwent a delay in erythroid progression starting at D10 with 11.6% of cells in R3 as compared with 53.5% for pPWI control cells. At D13 and D16, FIS1 OX cells displayed 21.5 and 22.0% of R3 cells, respectively as compared with 52.2 and 74.1% of R3 cells in control cells. Additionally, at D16, FIS1 OX cells displayed 38.2% R2 cells as compared with 0.97% R2 cells in control cells. R3 population is characterized by the onset of hemoglobin biosynthesis. On the other hand, FIS1 KD cells showed a normal differentiation pattern as compared with pLVCTH control cells (Supplementary Figure S3). These results suggest that exacerbation of mitochondrial fission at the early stages of erythroid differentiation disrupts hemoglobin biosynthesis and delays erythroid progression.

### Effects on Mitochondrial Parameters

**FIGURE 3:**
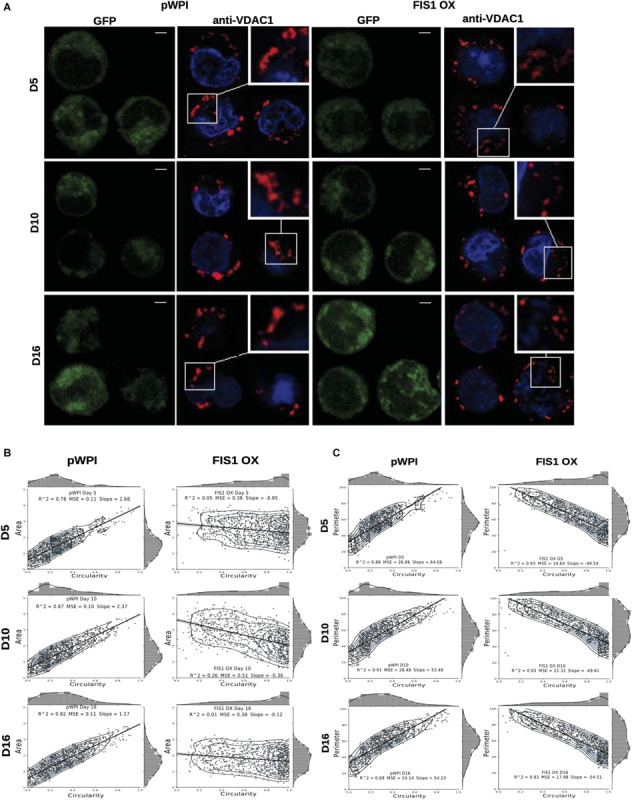
Effects of FIS1 OX on mitochondrial morphology. **(A)** Mitochondria visualization by confocal microscopy. Immunofluorescence microscopy at D5, D10, and D16 of erythroid differentiation for pWPI control and FIS1 OX cells. Anti-VDAC1 antibody labeled mitochondria (red) and the DAPI dye, the nucleus (blue). **(B)** Mitochondrial morphometric analysis in term of Area at D5, D10, and D16 of differentiation for control pWPI and FIS1 OX cells was performed using the Z-slides from GFP+ cells. Each dot represents a mitochondrial unit in terms of Area (*Y* axes) and Circularity (*X* axes). Also, a frequency histogram of mitochondrial circularity and area were added. Linear regression analysis was performed to compare slopes, which were significantly different (*p* < 0.05). R^2^ and MSE were also calculated **(C)** similar to panel **(B)**. Mitochondrial morphometric analysis in term of Perimeter. Each dot represents a mitochondrial unit in terms of Perimeter (*Y* axes) and circularity (*X* axes).

Assessment of mitochondrial morphology was performed in FIS1 OX cells (Figure 3) and FIS1 KD cells (Supplementary Figure S4) from D5 to D16 of differentiation by immuno-staining with anti-VDAC1 antibody (red channel). Mid-sagittal optical sections of positive-transduced cells (GFP+) were analyzed by confocal microscopy. As expected, FIS1 OX cells displayed a fragmented mitochondrial web with some large and round mitochondria as compared with pWPI control cells (Figure 3A). Mitochondrial morphology analysis in terms of circularity, area and perimeter revealed a very well-structured morphological pattern during erythroid differentiation in control cells. A positive correlation (positive slope) was found between circularity and area (Figure 3B); and between circularity and perimeter (Figure 3C) with a correlation coefficient (R^2^) of 0.78–0.87 and 0.88–0.91, respectively. Mitochondrial area (Figure 3B) and perimeter (Figure 3C) distribution (seen on the right-*Y* axis) shifted slightly toward smaller values during differentiation and circularity distribution (seen on the upper-*X* axis) ranged from 0 to 0.5 having a peak of 0.2 in control cells. The overexpression of FIS1 caused major changes in mitochondrial morphology which were evident as early as D5 and throughout erythroid differentiation as compared with control cells. Firstly, the correlation between circularity and area is lost with R^2^ values of 0.01 to 0.26 mainly because circularity distribution shifted considerably toward higher values (ranging from 0.6 to 1 with a peak of 0.9) with mitochondria having a larger area than control cells (Figure 3B). Furthermore, a negative correlation (negative slope) was found between circularity and perimeter with an R^2^ of 0.93; and the perimeter switched from normal to bimodal distribution at D10 (Figure 3C). These quantitative analyses of mitochondrial morphology for FIS1 OX cells are in agreement with the presence of a fragmented mitochondrial web with a greater amount of large and round mitochondria and suggest that unbalanced MtDy toward mitochondrial fission are responsible for disruption of both heme biosynthesis and erythroid progression. Regarding FIS1 KD cells, no differences were found at D5 and D10 as compared with controls. However, at D16, FIS1 KD cells have decreased mitochondrial circularity, area and perimeter (Supplementary Figure S4). Since FIS1 silencing did not affect hemoglobin biosynthesis or erythroid progression at least to the stage of polychromatophilic and orthochromatophilic erythroblast (R4-R5 as seen in Supplementary Figure S3), it will not be studied further.

**FIGURE 4:**
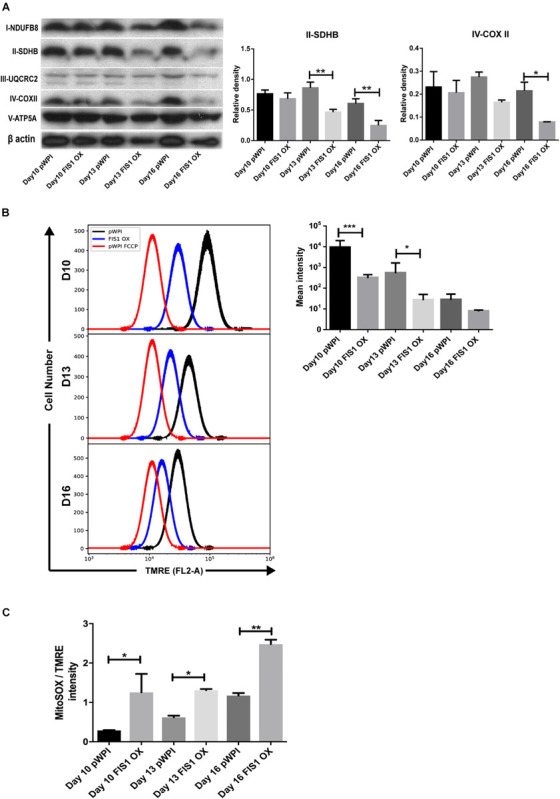
Effects of FIS1 OX on mitochondrial bioenergetics. **(A)** OXPHOS protein expression levels. Total protein lysate from FIS1 OX and pWPI control cells at D10, D13, and D16 of erythroid differentiation were assayed for immunoblot to detect the OXPHOS protein expression level. Band densitometry analysis was performed for Complex II and Complex IV. The plotted values are ±SEM. *n* = 3. Statistical analysis, ANOVA followed by Bonferroni *post hoc* test (* *p* < 0.05, **p < 0.01). **(B)** Measurement of mitochondrial membrane potential. FIS1 OX and pWPI control cells at D10, D13, and D16 erythroid differentiation were stained with 10 nM TMRE in non-quenching mode and analyzed by flow cytometry. 10 uM FCCP was used as negative control. TMRE mean intensity was quantified. The values plotted are ±SEMs. *n* = 3. Statistical analysis, ANOVA followed by Bonferroni *post hoc* test (* *p* < 0.05, *** *p* < 0.001). **(C)** Measurement of mitochondrial superoxide radical. FIS1 OX and pWPI control cells at D10, D13, and D16 erythroid differentiation were stained with 5 μM MitoSOX and analyzed by flow cytometry. MitoSOX mean intensity was quantified and the ratio MitoSOX/TMRE was calculated to obtain a normalized value of the amount of ROS between the cell groups, because MitoSOX uptake is dependent on mitochondrial membrane potential. The values plotted are ±SEMs. *n* = 3. Statistical analysis, ANOVA followed by Bonferroni *post hoc* test (*p < 0.05, **p < 0.01).

To gain insights into the bioenergetic capacity of mitochondria in FIS1 OX cells, OXPHOS protein expression, mitochondrial membrane potential and mitochondrial ROS were assessed at D10, D13 and D16 (Figure 4). At the level of protein expression, complex II significantly decreased by 50% (*p* < 0.01) at D13 and D16; and complex IV by 54% (*p* < 0.05) at D16 as compared with control pWPI cells (Figure 4A). As expected from the protein expression levels, mitochondrial membrane potential decreased 20% in FIS1 OX cells at D10 (*p* < 0.001) and 13 (*p* < 0.05) (Figure 4B) and no significant changes were observed at D16. However, FIS1 OX cells display twice more mitochondrial superoxide than control cells at D10 and D13 with a *p* < 0.05 and D16 with *p* < 0.01 even with a lower mitochondrial membrane potential (Figure 4C). These results clearly suggest that disruption of MtDy by FIS1 over-expression decreases the bioenergetic capacity of mitochondria.

### Effect of FIS1 OX on Cell Survival During Erythroid Differentiation

**FIGURE 5:**
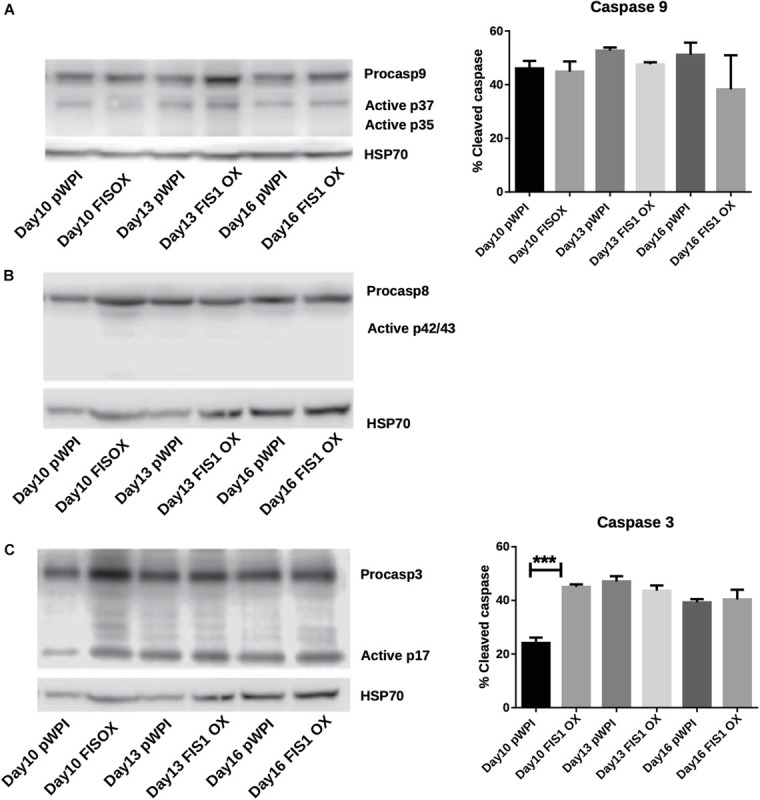
Assessment of apoptosis in FIS1 OX cells. Total protein lysates from FIS1 OX and pWPI control cells at D8, D10, D13, and D16 of erythroid differentiation were examined by Western blot. **(A)** Protein levels of Procaspase 9 and cleaved active forms P37 and P35. This is for intrinsic apoptosis. HSP70 were used as housekeeping control. Band densitometry for p37, as a percentage of total caspase 9, was plotted. Values are ±SEM. *n* = 3. Statistical analysis, ANOVA followed by Bonferroni *post hoc* test. **(B)** Protein levels of Procaspase 8 and cleaved active forms P42/P43. This is for extrinsic apoptosis. HSP70 were used as housekeeping control. **(C)** Protein levels of Procaspase 3 and cleaved active form P17. This is a general apoptotic effector. HSP70 were used as housekeeping control. Band densitometry for p17, as a percentage of total caspase 3, was plotted. Values are ±SEM. *n* = 3. Statistical analysis, ANOVA followed by Bonferroni *post hoc* test (*** *p* < 0.001).

Our results demonstrate a loss of mitochondrial homeostasis that could lead to programmed cell death. Expression of apoptosis markers was measured to determine whether apoptosis was responsible for the disruption in hemoglobin biosynthesis and delayed erythropoiesis in FIS1 OX cells. By Western blot, the levels of pro-apoptotic active caspase 9 (intrinsic pathway), active caspase 8 (extrinsic pathway), and active caspase 3 (apoptosis executioner) were measured in differentiating FIS1 OX and control cells at D10, D13, and D16 (Figure 5). No significant differences were found in cleaved caspase 9 (Figure 5A) and caspase 8 (Figure 5B). While caspase 3 showed an initial difference at Day 10 in FIS1 OX, this was not sustained throughout the following days (Figure 5C). Caspases have been reported to play a role in erythropoiesis, in particular, caspase 3 (Boehm et al., 2013) and that may explain the presence of active proteins. However, since no differences were observed between FIS1 OX and control cells, these results suggest that apoptosis is not triggered by FIS1 overexpression and, therefore, is not involved in the hemoglobin and erythropoiesis disruption seen in FIS1 OX cells.

### MFN1 Knock-Down (MFN1 KD) Delays Erythroid Differentiation

**FIGURE 6:**
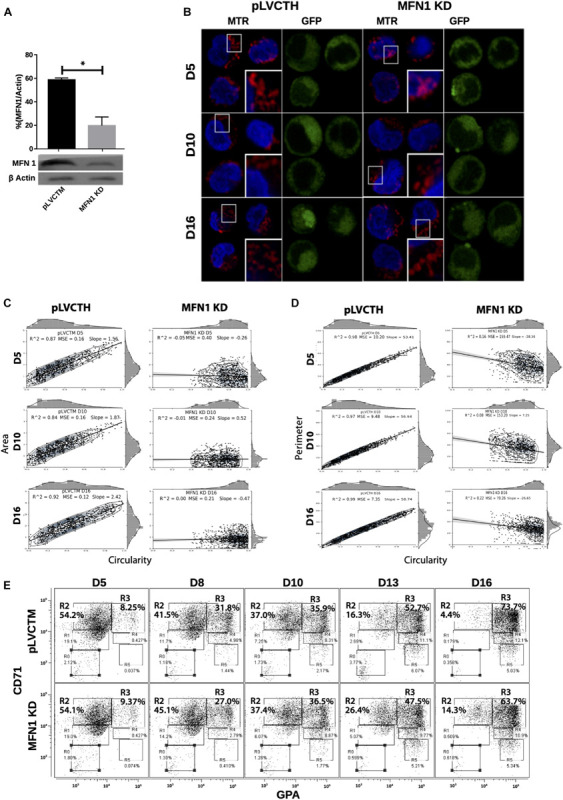
Effect of MFN1 KD on mitochondrial morphology and erythroid differentiation. **(A)** Western blot to detect the knock-down of MFN1. Total protein lysates from pLVCTH control and MFN1 KD cells at D3 of erythroid differentiation were examined by Western blot to check MFN1 expression. Quantification was performed by band densitometry. Plotted values are ±SEM. *n* = 3. Statistical analysis, ANOVA followed by Bonferroni *post hoc* test (* *p* < 0.05). **(B)** Mitochondria visualization by confocal microscopy. Mitochondria were stained with 10 nM MitoTracker Red CMXRos and visualized at D5, D8, D10, D13, and D16 of erythroid differentiation for pLVCTH control and MFN1 KD cells. The nucleus was stained with DAPI (blue). **(C)** Mitochondrial morphometric analysis in term of Area at D5, D10, and D16 of erythroid differentiation for pLVCTH control and MFN1 KD cells. It was performed using the Z-slides from GFP+ cells. Each dot represents a mitochondrial unit in terms of area (*Y* axes) and circularity (*X* axes). Also, a frequency histogram of mitochondrial circularity and area were added. Linear regression analysis was performed to compare slopes, which were significantly different (*p* < 0.05). R^2^ and MSE were also calculated. **(D)** Similar to panel **(C)**. Mitochondrial morphometric analysis in term of Perimeter. Each dot represents a mitochondrial unit in terms of Perimeter (*Y* axes) and circularity (*X* axes). **(E)** Erythroid progression in MFN1 KD cells. Flow cytometry analysis of surface markers anti-CD71-APC and anti-GPA-PE in pLVCTH control and MFN1 KD cells at D5, D10, and D16 days of EPO-induced differentiation. All analysis (*n* = 3) correspond GFP+ cells. MFN1 KD cells have a bigger R2 and smaller R3 population at D13 and D16 than control cells, meaning a delay in erythroid progression as compared with control cells.

To confirm whether a MtDy is a main actor in the erythropoietic cell differentiation, MFN1 was knocked down to mimic the FIS1 OX phenotype i.e., the fragmentation of the mitochondrial web and the appearance of large and round mitochondria. It has been reported that MFN1 knock-down (MFN1 KD) triggers round mitochondria morphology in several cell models (Chen et al., 2003; Hall et al., 2016; Yamada et al., 2017). Immediately after isolation, CD34+ cells were transduced with pLVCTM-shMFN1 lentiviral particles to knock-down MFN1 protein. MFN1 depletion was confirmed by Western blot, which showed a significant 58% reduction in MFN1 protein levels (*p* < 0.001) (Figure 6A). Mitochondria-morphological analysis of MFN1 KD cells revealed a distinct pattern as compared with FIS1 OX cells. MFN1 KD cells displayed a fragmented but homogeneous mitochondria population in terms of area, perimeter and circularity during erythroid differentiation (Figure 6) as compared with FIS1 OX cells (Figure 3). MFN1KD mitochondria exhibited mainly a reduction in size and an increase in circularity i.e., mitochondria became rounded and smaller than control cells. Comparing FIS1 OX with MFN1 KD mitochondria population, the former is an average of three times bigger than the latter (Figure 6C versus Figure 3B). Erythroid progression analysis by flow cytometry showed that MFN1 KD caused a delay in erythroid progression but did not disrupt heme biosynthesis. This was evident at D16 comparing the R2 and R3 populations. R2 cells were 4.14% for control cells and 14.3% for MFN1 KD cells; and R3 cells, 73.7% for controls cells and 63.7% for MFN1 KD cells (Figure 6E).

Even though FIS1 overexpression and MFN1 knockdown resulted in mitochondrial web fragmentation, the final output of the mitochondrial phenotype was distinctive and associated with divergent physiological consequences in erythropoiesis. These interesting results suggest that the larger mitochondrial size in FIS1 OX mitochondria might be the consequence of another mechanism coupled to MtDy through the action of FIS1 protein.

### Closure of the Mitochondrial Permeability Transition Pore (mPTP) Rescues the Erythroid Differentiation in FIS1 OX Cells

**FIGURE 7:**
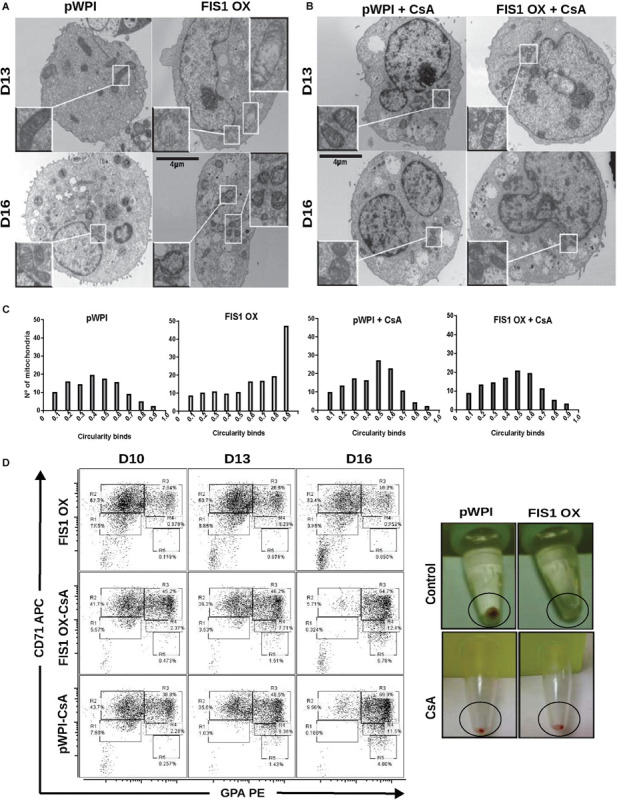
Assessment of the mPTP in FIS1 OX cells. **(A)** Ultrastructure visualization of mitochondria. Transmission electron microscopy (TEM) was performed for pWPI control and FIS1 OX cells at D13 and D16 of erythroid differentiation. FIS1 OX displayed heterogeneous mitochondrial morphologies. Some mitochondria look like immature mitochondria with short and few cristae. It was also found swollen-like mitochondria as compared with control cells. Magnification 6000×. **(B)** Ultrastructure visualization of mitochondria in CsA-treated cells. TEM analysis showed normal mitochondrial structure and electron density in CsA-treated FIS1 OX cells as compared with CsA-treated pWPI control cells. **(C)** Mitochondrial circularity analysis. It was calculated from TEM images of pWPI control and FIS1 OX cells treated or not treated with CsA at D13 of erythroid differentiation. Frequency distributions showed that the treatment with CsA fully rescued the mitochondrial morphology. Data was analyzed using contingency tables and a Chi-square test (*p* < 0.05). **(D)** Erythroid progression analysis of CsA-treated FIS1 OX cells. Cells were treated with CsA on D9 and collected at D10, D13, and D16. Right after collection, cells were immunolabeled with anti-CD71-APC and anti-GPA-PE and analyzed by flow cytometry. FIS1 OX cells with no CsA treatment are also shown. All analysis (*n* = 3) correspond to GFP+ cells. In addition, cells were spun down to observe the cell pellet’s color. Treatment with CsA rescued erythroid differentiation and hemoglobin biosynthesis in FIS1 OX cells.

The appearance of large and round mitochondria with low membrane potential and reduced OXPHOS expression, along with the disruption of heme biosynthesis and the cessation of erythropoiesis in FIS1 OX cells, suggested the involvement of the mitochondrial permeability transition pore (mPTP). TEM was used to analyze mitochondrial ultrastructure and provide further insight into this process (Figure 7A). Control cells displayed mitochondria with normal morphology, matrix density and cristae structure at D13 and D16 of differentiation. On the other hand, FIS1 OX cells displayed a heterogeneous mitochondria population with immature-like mitochondria with few, poorly developed cristae and swollen-like mitochondria (Figure 7A). These ultrastructure images of mitochondria, which are in agreement with the morphology results obtained by confocal microscopy (Figure 3), suggested the involvement of the mPTP as a potential mechanism of signaling between MtDy and the erythropoietic phenotype. To corroborate this, FIS1 OX and control cells were treated with the mPTP inhibitor cyclosporin A (CsA). TEM images corroborated that CsA treatment rescued mitochondrial morphology. CsA-treated FIS1 OX cells had mitochondria with normal size and cristae structure similar to control cells (Figure 7B); and the quantitative analysis of mitochondrial circularity (Figure 7C) confirmed the recovery in CsA-treated FIS1 OX cells. Finally, flow cytometry analysis of cells collected at D10, D13, and D16 of erythroid differentiation also showed that CsA treatment rescued erythroid maturation and heme biosynthesis in FIS1 OX cells. In addition, CsA-treated FIS1 OX cells have a similar differentiation pattern that control cells (Figure 7D). Our findings suggest that changes in MtDy toward a more fragmented mitochondrial web due to FIS1 overexpression correlates with the opening of the mPTP, which in turn arrested erythroid differentiation and inhibited hemoglobin biosynthesis.

## Discussion

Fine regulation of mitochondrial fission and fusion is required for energy demands adjustments, metabolic control and signaling, allowing proper cellular function (Wai and Langer, 2016) cell proliferation and differentiation (Karbowski, 2002; Arciuch et al., 2012), stem cell commitment (Forni et al., 2016) and cell death. In this work, we contribute to the understanding of MtDy during erythropoiesis. We found that the most expressed fission genes are *fis1* and *drp1*; and the fusion gene most highly expressed was *mfn1*. All of these genes have a well-defined expression pattern, increasing their mRNA levels as erythroid differentiation moves forward. Furthermore, we showed that erythropoiesis is dependent on a shift in the balance of MtDy, where progression from progenitors to proerythroblast is characterized by increased levels of mitochondrial fusion and decreased fission. Balance displacement toward fission by means of FIS1 overexpression at early stages, disrupted mitochondrial morphology and function, heme biosynthesis and delayed erythroid differentiation, keeping cells in an undifferentiated state. Our results also suggested a link between mitochondrial fusion and fission events and the opening/closure of the mPTP, establishing a potential mechanism for MtDy signaling.

It has been reported that HSC have a glycolytic metabolism and when they are induced to differentiate, they switch to oxidative metabolism supported by increased mitochondrial biomass, cristae maturation, increased OXPHOS protein density and elongated (fused) mitochondria. All of these changes contribute to increased ROS levels, which acts as a second messenger to drive cellular differentiation through modification of the transcriptional landscape, which controls proliferation or differentiation (Piccoli et al., 2013; Tatapudy et al., 2017; Laaper and Jahani-Asl, 2018; Khacho et al., 2019; Samimi et al., 2020). Therefore, fusion impairment may affect proper mitochondria maturation, OXPHOS density and then the production of ROS and ATP. Disruption of mitochondrial fusion by MFN1 KD also delays erythroid progression. Several studies about cell reprogramming have shown that depletion of MFN1 and MFN2, facilitates the conversion of somatic cells to pluripotent cells, decreases the expression of OXPHOS subunits and promotes glycolytic metabolism through the involvement of the hypoxia-inducible factor 1-α (HIF1α) (Son et al., 2015). Also, depletion of MFN1 in mouse embryonic heart cells stalled the heart development through impairments in cell differentiation to cardiomyocytes (Kasahara et al., 2013; Noguchi and Kasahara, 2017; Yamada et al., 2018). The role of MtDy in commitment and differentiation of stem cells has been also shown in neural stem cells for neurogenesis (Khacho et al., 2016; Khacho and Slack, 2018). These evidences strengthen our results that changing the balance of MtDy toward a more fragmented state is able to induce a metabolic reprogramming affecting the differentiation process.

The role of FIS1 as a direct receptor of DRP1 to induce mitochondrial fission has been controversial in mammals because recruitment of DRP1 to mitochondria or DRP1-mediated fission has been shown to be independent of FIS1 expression levels (Otera et al., 2010, 2013; Losón et al., 2013). In fact, it was recently published that FIS1 is an inhibitor of mitochondrial fusion, interacting directly with MFN1, MFN2, and OPA1 (Yu et al., 2019). Thus, mitochondria fragmentation in FIS1 overexpressing cells would be due to the inhibition of mitochondrial fusion. However, other recent papers have shown the efficacy of a molecule able to inhibit the interaction DRP1/FIS1, which blocks pathological or excessive mitochondrial fragmentation seen in ALS and cardiac diseases without affecting basal mitochondrial fission (Haileselassie et al., 2019). Our results showed that both FIS1 OX and MFN1 KD erythropoietic cells had delayed erythropoiesis. Nevertheless, this effect was more evident and stronger in FIS1 OX cells, and only FIS1 OX cells displayed a disruption in heme biosynthesis. Furthermore, the mitochondrial web fragmentation caused by FIS1 OX and MFN1 KD are not alike as revealed by a deep mitochondrial morphological analysis. FIS1 OX cells had a fragmented mitochondrial network but with heterogeneous and bigger mitochondria (large and round mitochondria) than MNF1 KD cells, which also have fragmented mitochondria, but they are homogeneous and smaller. Our data suggest that both FIS1 OX and MFN1 KD are able to cause a mitochondrial web fragmentation, but they do not have a redundant role in mitochondrial function in erythropoiesis. Regarding FIS1 KD cells, erythropoiesis was not altered at least to the level of polychromatophilic and orthochromatophilic erythroblasts (R4 and R5 population). However, changes in mitochondrial morphology were observed at D16. Thus, our results cannot exclude a role for FIS1 from orthochromatophilic erythroblast to fully mature erythrocyte, especially considering those maturation stages are characterized by extensive mitophagy which is dependent on mitochondrial fragmentation to get rid of all mitochondria (Betin et al., 2013; Yamashita et al., 2016).

Excessive mitochondrial fission has been associated with a decreased mitochondrial function and increased ROS (Jheng et al., 2011; Haileselassie et al., 2019). FIS1 up-regulation decreased cellular ATP levels in anoxic cardiomyocytes and impaired glucose-stimulated insulin secretion in INS-1E cells (Park et al., 2008; Wang et al., 2012); and it has been identified as a molecular marker for poor prognosis in patients with acute myeloid leukemia (Tian et al., 2014). We observed that erythroid differentiation under FIS1 overexpression was arrested mainly at the proerythroblast level with mitochondria featuring reduced complex II and IV protein expression and membrane potential, increased ROS, and an immature phenotype i.e., short and poor developed cristae as seen by TEM. No significant dead or apoptotic cells were observed, which suggested that FIS1 overexpression induced mitochondrial metabolic reprogramming that maintains cells in the glycolytic state to support cell proliferation rather than shift to oxidative metabolism associated with cell maturation. Furthermore, our results support the evidence of excessive levels of FIS 1 as a molecular marker of myeloid leukemia (Tian et al., 2014). Interestingly, mitochondrial fragmentation by knocking down MFN1 or OPA1 increases ROS levels without inducing any signs of oxidative damage or cell viability (Laaper and Jahani-Asl, 2018).

At this point, we have a paradoxical situation where exacerbation of fission by FIS1 overexpression caused increased ROS, normally associated with cellular differentiation and maturation, but erythroid progression was arrested. Additionally, how are ROS increased when the mitochondrial membrane potential is low and respiratory complex II and IV are reduced? Published literature suggests that reduction in the respiratory complexes II and IV, but not in the others, generates a bottleneck for electron transport and increases electron leaking and then ROS generation. It is also notable that inhibition of complex II not only affect ETC but also the TCA cycle, causing increased succinate levels which inhibit the Prolyl-Hydroxylases to allow the condition called pseudohypoxia through the stabilization of HIF1α stabilization (Grimolizzi and Arranz, 2018; Kluckova and Tennant, 2018) and launch a metabolic reprogramming toward more immature cells and mitochondria. In this regard, Ansó et al. (2017) showed that the ablation of Complex III in mouse HSC allows them to proliferate but impairs their differentiation. The mechanisms involved a decreased NAD+/NADH ratio and increased 2-hydroxyglutarate which is known to regulate transcriptional and epigenetics cell programs. Furthermore, the opening of mPTP may result in a loss of antioxidant molecules and promote an increase in ROS. Furthermore, it reduces membrane potential and impairs electron transport due to the disruption of respiratory complex II and I in cardiomyocytes (Hom et al., 2011; Folmes et al., 2012).

Although the role of MtDy for cell physiology is well accepted, there is no clarity about the signaling mechanisms to induce metabolic reprogramming which involves mitochondria-nucleus retrograde communication and transcriptional regulation. Our results suggest that the mPTP, is a link between MtDy and the signaling mechanisms to control cell differentiation. FIS1 OX generated large and round mitochondria, which were not seen in MFN1 KD cells. Treatments with cyclosporin A (CsA), which is an inhibitor of the mPTP, rescued the mitochondrial morphology, the heme biosynthesis and the progression of erythropoiesis. Interestingly, the role of the mPTP in cellular differentiation has been previously reported for early embryonic cardiomyocytes, where a frequent opening of the mPTP is needed to maintain an immature mitochondrial morphology with low oxidative capacity but also elevated ROS, which is reduced upon the stimulated mPTP closure. Closure of the pore was also required for proper differentiation into cardiac cells (Hom et al., 2011; Folmes et al., 2012). Furthermore, mPTP flashes are needed for cortical neural progenitor differentiation (Hou et al., 2013); and closure of mPTP with CsA is protective against extra physiologic oxygen shock/stress in HSC transplantation (Mantel et al., 2015); and needed for a more efficient *in vitro* differentiation of CD34^+^ cells to red blood cells (Ronzoni et al., 2008).

One of the triggers of mPTP opening is an elevated Ca^++^ concentration in the mitochondrial matrix (Bernardi and Di Lisa, 2015). In this regard, FIS1 can interact with the ER protein BAP31 allowing mitochondria to be loaded with Ca^++^ (Iwasawa et al., 2011; Wang et al., 2011) and then establishing a potential mechanism between MtDy and the mPTP. The role of mitochondrial fusion at early stages of erythropoiesis, according to our results, is to keep the mPTP closed, engage OXPHOS for ATP synthesis and proper ROS generation to enable the correct execution of the transcriptional program required to drive cell differentiation.

In summary, the proper balance of MtDy is essential for erythroid differentiation. Disruption of MtDy by FIS1 overexpression in HSC altered mitochondrial function, delayed their differentiation into red blood cells and blocked heme biosynthesis, keeping them in an immature and proliferative state. The red blood cell differentiation in FIS1 OX cells was fully rescued by the addition of CsA, a pharmacological inhibitor of the mPTP, into the culture media. We have also shown that MFN1 KD caused mitochondrial fragmentation and delayed erythropoiesis but did not caused the same effects on mitochondrial morphology as FIS OX and did not block heme biosynthesis. Thus, MFN1 and FIS1 are not redundant in their functions. Our results suggest that MtDy regulates the opening/closure of the mPTP, which in turn will allow/block the exit of signal molecules such a Ca^++^, ROS and other metabolites to manage the genetic and metabolic reprogramming of cells for proper cellular differentiation.

## Data Availability Statement

The raw data supporting the conclusions of this article will be made available by the authors, without undue reservation.

## Ethics Statement

The studies involving human participants were reviewed and approved by the Scientific Ethics Committee of the South Metropolitan Health Service (SSMS), Ministry of Health, Chile; and the Ethics Committee of Andres Bello University, Chile. Both Committees subscribe to the Declaration of Helsinki. The patients/participants provided their written informed consent to participate in this study.

## Author Contributions

AG-I performed most of the experiments and data analysis and contributed with manuscript writing. LR and EJ performed TEM analyses. CE and VR performed apoptosis experiments. LS and OS designed and performed the qPCR on G1E-ER cells. AE conceived the research project, made the experimental design, analyzed the data, wrote the manuscript, and funded the research. All authors contributed to the article and approved the submitted version.

## Conflict of Interest

The authors declare that the research was conducted in the absence of any commercial or financial relationships that could be construed as a potential conflict of interest.

## Supplementary Material

Supplementary Figure 1 Mitochondrial dynamics in mouse erythropoiesis. **(A)** Visualization of mitochondrial morphology. The mouse erythropoietic cell line G1E-ER were β-estradiol-treated to induced erythroid differentiation. Mitochondria were stained with 10 nM MitoTracker Red CMXRos and visualized by confocal microscopy at 24 h post induction. It is shown one cell per condition. The mitochondrial web increased its biomass and became fragmented upon erythroid differentiation. **(B)** Mitochondrial Dynamics gene expression. mRNA expression kinetics of mitochondrial dynamics genes *Fis1*, *Opa1*, *Mnf1*, *Mfn2* during β-estradiol-induced erythropoiesis in G1E-ER cells, was assessed by quantitative real time (qRT-PCR). Cells were collected at 0, 12, 24, 36, and 48 h. The left panel shows the all four genes and the right panel, just *Mfn1* and *Mfn2* genes at higher scale. Average expression values are ±STD of *n* = 3. Statistical analysis was ANOVA followed by Bonferroni post hoc test (*p* < 0.05).

Supplementary Figure 2 Lentiviral transduction of CD34+ cells. CD34+ cells were lentivirus-transduced one hour after isolation overnight. 3 days later, transduction efficiency was assessed by flow cytometry. GFP expression was used as indicator of successful transduction. In addition, cells were stained with 7-AAD to assess dead cells. Hydrogen Peroxide was used in control cells as a positive control of dead cells. Transduction efficiency of CD34+ was over 90% with no dead cells (<1%).

Supplementary Figure 3 Erythroid Progression in FIS1 KD cells. Flow cytometry analysis of surface markers anti-CD71-APC and anti-GPA-PE in pLVCTH control and FIS1 KD cells at D5, D8, D10, D12, and D16 of EPO-induced erythroid differentiation (*n* = 3).

Supplementary Figure 4 Effects of FIS1 KD on mitochondrial morphology. **(A)** Mitochondria visualization by confocal microscopy. Immunofluorescence microscopy at D5, D10, and D16 of erythroid differentiation for pLVCTH control and FIS1 KD cells. Anti-VDAC1 antibody labeled mitochondria (red) and the DAPI dye, the nucleus (blue). **(B)** Mitochondrial morphometric analysis in term of Area at D5, D10, and D16 of differentiation for control pWPI and FIS1 KD cells. It was performed using the Z-slides from GFP+ cells. Each dot represents a mitochondrial unit in terms of Area (*Y* axes) and Circularity (*X* axes). Also, a frequency histogram of mitochondrial circularity and area were added. Linear regression analysis was performed to compare slopes, which were significantly different (*p* < 0.05). R^2^ and MSE were also calculated.

## References

[B1] Ahlqvist K. J. Suomalainen A. Hämäläinen R. H. (2015). Stem cells, mitochondria and aging. *Biochim. Biophys. Acta Bioenerget.* 1847 1380–1386. 10.1016/j.bbabio.2015.05.014 26014347

[B2] Ansó E. Weinberg S. E. Diebold L. P. Thompson B. J. Malinge S. Schumacker P. T. (2017). The mitochondrial respiratory chain is essential for haematopoietic stem cell function. *Nat. Cell Biol.* 19 614–625. 10.1038/ncb3529 28504706 PMC5474760

[B3] Arciuch V. G. A. Elguero M. E. Poderoso J. J. Carreras M. C. (2012). Mitochondrial regulation of cell cycle and proliferation. *Antioxid. Redox Signal.* 16 1150–1180. 10.1089/ars.2011.4085 21967640 PMC3315176

[B4] Bernardi P. Di Lisa F. (2015). The mitochondrial permeability transition pore: molecular nature and role as a target in cardioprotection. *J. Mol. Cell Cardiol.* 78 100–106. 10.1016/j.yjmcc.2014.09.023 25268651 PMC4294587

[B5] Betin V. M. S. Singleton B. K. Parsons S. F. Anstee D. J. Lane J. D. (2013). Autophagy facilitates organelle clearance during differentiation of human erythroblasts: evidence for a role for ATG4 paralogs during autophagosome maturation. *Autophagy* 9 881–893. 10.4161/auto.24172 23508006 PMC3672297

[B6] Bigarella C. L. Liang R. Ghaffari S. (2014). Stem cells and the impact of ROS signaling. *Development* 141 4206–4218. 10.1242/dev.107086 25371358 PMC4302918

[B7] Boehm D. Mazurier C. Giarratana M.-C. Darghouth D. Faussat A.-M. Harmand L. (2013). Caspase-3 is involved in the signalling in erythroid differentiation by targeting late progenitors. *PLoS One* 8:e62303. 10.1371/journal.pone.0062303 23658722 PMC3642196

[B8] Bonora M. Pinton P. Ito K. (2015). Mitochondrial control of hematopoietic stem cell balance and hematopoiesis. *Front. Biol.* 10 117–124. 10.1007/s11515-015-1356-0

[B9] Buck M. D. O’Sullivan D. Geltink R. I. K. Curtis J. D. Chang C.-H. Sanin D. E. (2016). Mitochondrial dynamics controls T cell fate through metabolic programming. *Cell* 166 63–76. 10.1016/j.cell.2016.05.035 27293185 PMC4974356

[B10] Campello S. Lacalle R. A. Bettella M. Mañes S. Scorrano L. Viola A. (2006). Orchestration of lymphocyte chemotaxis by mitochondrial dynamics. *J. Exp. Med.* 203 2879–2886. 10.1084/jem.20061877 17145957 PMC2118173

[B11] Chen H. Detmer S. A. Ewald A. J. Griffin E. E. Fraser S. E. Chan D. C. (2003). Mitofusins Mfn1 and Mfn2 coordinately regulate mitochondrial fusion and are essential for embryonic development. *J. Cell Biol.* 160 189–200. 10.1083/jcb.200211046 12527753 PMC2172648

[B12] Dailey H. A. Meissner P. N. (2013). Erythroid heme biosynthesis and its disorders. *Cold Spring Harb. Perspect. Med.* 3:a011676. 10.1101/cshperspect.a011676 23471474 PMC3683999

[B13] Folmes C. D. L. Dzeja P. P. Nelson T. J. Terzic A. (2012). Mitochondria in control of cell fate. *Circ. Res.* 110 526–529. 10.1161/RES.0b013e31824ae5c1 22343555 PMC3491643

[B14] Fontenay M. Cathelin S. Amiot M. Gyan E. Solary E. (2006). Mitochondria in hematopoiesis and hematological diseases. *Oncogene* 25 4757–4767. 10.1038/sj.onc.1209606 16892088

[B15] Forni M. F. Peloggia J. Trudeau K. Shirihai O. Kowaltowski A. J. (2016). Murine mesenchymal stem cell commitment to differentiation is regulated by mitochondrial dynamics. *Stem Cells* 34 743–755. 10.1002/stem.2248 26638184 PMC4803524

[B16] Gandre-Babbe S. van der Bliek A. M. (2008). The novel tail-anchored membrane protein mff controls mitochondrial and peroxisomal fission in mammalian cells. *Mol. Biol. Cell* 19 2402–2412. 10.1091/mbc.E07-12-1287 18353969 PMC2397315

[B17] Giarratana M. C. Kobari L. Lapillonne H. (2005). Ex vivo generation of fully mature human red blood cells from hematopoietic stem cells. *Nature* 23 69–74. 10.1038/nbt1047 15619619

[B18] Gonzalez-Ibanez A. M. Ruiz L. M. Jensen E. Echeverria C. A. Romero V. Stiles L. (2020). Exacerbation of mitochondrial fission in human CD34+ cells halts erythropoiesis and hemoglobin biosynthesis. *bioRxiv* [Preprint]. 10.1101/2020.07.31.230961

[B19] Grimolizzi F. Arranz L. (2018). Multiple faces of succinate beyond metabolism in blood. *Haematologica* 103 1586–1592. 10.3324/haematol.2018.196097 29954939 PMC6165802

[B20] Haileselassie B. Mukherjee R. Joshi A. U. Napier B. A. Massis L. M. Ostberg N. P. (2019). Drp1/Fis1 interaction mediates mitochondrial dysfunction in septic cardiomyopathy. *J. Mol. Cell Cardiol.* 130 160–169. 10.1016/j.yjmcc.2019.04.006 30981733 PMC6948926

[B21] Hall A. R. Burke N. Dongworth R. K. Kalkhoran S. B. Dyson A. Vicencio J. M. (2016). Hearts deficient in both Mfn1 and Mfn2 are protected against acute myocardial infarction. *Cell Death Dis.* 7:e2238. 10.1038/cddis.2016.139 27228353 PMC4917668

[B22] Held N. M. Houtkooper R. H. (2015). Mitochondrial quality control pathways as determinants of metabolic health. *Bioessays* 37 867–876. 10.1002/bies.201500013 26010263 PMC5053262

[B23] Hom J. R. Quintanilla R. A. Hoffman D. L. de Mesy Bentley K. L. Molkentin J. D. Sheu S.-S. (2011). The permeability transition pore controls cardiac mitochondrial maturation and myocyte differentiation. *Dev. Cell* 21 469–478. 10.1016/j.devcel.2011.08.008 21920313 PMC3175092

[B24] Hou Y. Mattson M. P. Cheng A. (2013). Permeability transition pore-mediated mitochondrial superoxide flashes regulate cortical neural progenitor differentiation. *PLoS One* 8:e76721. 10.1371/journal.pone.0076721 24116142 PMC3792897

[B25] Iwasawa R. Mahul-Mellier A.-L. Datler C. Pazarentzos E. Grimm S. (2011). Fis1 and Bap31 bridge the mitochondria-ER interface to establish a platform for apoptosis induction. *EMBO J.* 30 556–568. 10.1038/emboj.2010.346 21183955 PMC3034017

[B26] Jensen E. L. Gonzalez-Ibanez A. M. Mendoza P. Ruiz L. M. Riedel C. A. Simon F. (2019). Copper deficiency-induced anemia is caused by a mitochondrial metabolic reprogramming in erythropoietic cells. *Metallomics* 11 282–290. 10.1039/C8MT00224J 30358789

[B27] Jheng H. F. Tsai P. J. Guo S. M. Kuo L. H. Chang C. S. Su I. J. (2011). Mitochondrial Fission Contributes to Mitochondrial Dysfunction and Insulin Resistance in Skeletal Muscle. *Mol. Cell. Biol.* 32 309–319. 10.1128/MCB.05603-11 22083962 PMC3255771

[B28] Karbowski M. (2002). Spatial and temporal association of Bax with mitochondrial fission sites, Drp1, and Mfn2 during apoptosis. *J. Cell Biol.* 159 931–938. 10.1083/jcb.200209124 12499352 PMC2173996

[B29] Kasahara A. Cipolat S. Chen Y. Dorn G. W. Scorrano L. (2013). Mitochondrial fusion directs cardiomyocyte differentiation via calcineurin and Notch signaling. *Science* 342 734–737. 10.1126/science.1241359 24091702

[B30] Khacho M. Clark A. Svoboda D. S. Azzi J. MacLaurin J. G. Meghaizel C. (2016). Mitochondrial Dynamics Impacts Stem Cell Identity and Fate Decisions by Regulating a Nuclear Transcriptional Program. *Stem Cell* 19 1–17. 10.1016/j.stem.2016.04.015 27237737

[B31] Khacho M. Harris R. Slack R. S. (2019). Mitochondria as central regulators of neural stem cell fate and cognitive function. *Nat. Rev. Neurosci.* 20 34–48. 10.1038/s41583-018-0091-3 30464208

[B32] Khacho M. Slack R. S. (2018). Mitochondrial dynamics in the regulation of neurogenesis: from development to the adult brain. *Dev. Dyn.* 247 47–53. 10.1002/dvdy.24538 28643345

[B33] Kluckova K. Tennant D. A. (2018). Metabolic implications of hypoxia and pseudohypoxia in pheochromocytoma and paraganglioma. *Cell Tissue Res.* 1–12. 10.1007/s00441-018-2801-6 29450727 PMC5915505

[B34] Kluge M. A. Fetterman J. L. Vita J. A. (2013). Mitochondria and endothelial function. *Circ. Res.* 112 1171–1188. 10.1161/CIRCRESAHA.111.300233 23580773 PMC3700369

[B35] Laaper M. Jahani-Asl A. (2018). Regulation of neural stem cell fate decisions by mitochondrial dynamics. *Neural Regen. Res.* 13 1548–1549. 10.4103/1673-5374.237115 30127113 PMC6126118

[B36] Liesa M. Shirihai O. S. (2013). Mitochondrial dynamics in the regulation of nutrient utilization and energy expenditure. *Cell Metab.* 17 491–506. 10.1016/j.cmet.2013.03.002 23562075 PMC5967396

[B37] Losón O. C. Song Z. Chen H. Chan D. C. (2013). Fis1, Mff, MiD49, and MiD51 mediate Drp1 recruitment in mitochondrial fission. *Mol. Biol. Cell* 24 659–667. 10.1091/mbc.E12-10-0721 23283981 PMC3583668

[B38] Mantel C. R. O’Leary H. A. Chitteti B. R. Huang X. Cooper S. Hangoc G. (2015). Enhancing hematopoietic stem cell transplantation efficacy by mitigating oxygen shock. *Cell* 161 1553–1565. 10.1016/j.cell.2015.04.054 26073944 PMC4480616

[B39] Maryanovich M. Gross A. (2013). A ROS rheostat for cell fate regulation. *Trends Cell Biol.* 23 129–134. 10.1016/j.tcb.2012.09.007 23117019

[B40] Moras M. Lefevre S. D. Ostuni M. A. (2017). From erythroblasts to mature red blood cells: organelle clearance in mammals. *Front. Physiol.* 8:1076. 10.3389/fphys.2017.01076 29311991 PMC5742207

[B41] Noguchi M. Kasahara A. (2017). Mitochondrial dynamics coordinate cell differentiation. *Biochem. Biophys. Res. Commun.* 500 59–64. 10.1016/j.bbrc.2017.06.094 28634072

[B42] Osti F. Corradini F. G. Hanau S. Matteuzzi M. Gambari R. (1997). Human leukemia K562 cells: induction to erythroid differentiation by guanine, guanosine and guanine nucleotides. *Haematologica* 82 395–401. 9299849

[B43] Otera H. Ishihara N. Mihara K. (2013). New insights into the function and regulation of mitochondrial fission. *Biochim. Biophys. Acta Mol. Cell Res.* 1833 1256–1268. 10.1016/j.bbamcr.2013.02.002 23434681

[B44] Otera H. Wang C. Cleland M. M. Setoguchi K. Yokota S. Youle R. J. (2010). Mff is an essential factor for mitochondrial recruitment of Drp1 during mitochondrial fission in mammalian cells. *J. Cell Biol.* 191 1141–1158. 10.1083/jcb.201007152 21149567 PMC3002033

[B45] Park K.-S. Wiederkehr A. Kirkpatrick C. Mattenberger Y. Martinou J.-C. Marchetti P. (2008). Selective actions of mitochondrial fission/fusion genes on metabolism-secretion coupling in insulin-releasing cells. *J. Biol. Chem.* 283 33347–33356. 10.1074/jbc.M806251200 18832378 PMC2662262

[B46] Piccoli C. Agriesti F. Scrima R. Falzetti F. Di Ianni M. Capitanio N. (2013). To breathe or not to breathe: the haematopoietic stem/progenitor cells dilemma. *Br. J. Pharmacol.* 169 1652–1671. 10.1111/bph.12253 23714011 PMC3753828

[B47] Pourcelot M. Arnoult D. (2014). Mitochondrial dynamics and the innate antiviral immune response. *FEBS J.* 281 3791–3802. 10.1111/febs.12940 25051991

[B48] Prieto J. Torres J. (2017). Mitochondrial dynamics: in cell reprogramming as it is in cancer. *Stem Cells Int.* 2017 8073721. 10.1155/2017/8073721 28484497 PMC5412136

[B49] Ronzoni L. Bonara P. Rusconi D. Frugoni C. Libani I. Cappellini M. D. (2008). Erythroid differentiation and maturation from peripheral CD34+ cells in liquid culture: cellular and molecular characterization. *Blood Cells Mol. Dis.* 40 148–155. 10.1016/j.bcmd.2007.07.006 17889571

[B50] Ruiz L. M. Jensen E. L. Rossel Y. Puas G. I. Gonzalez-Ibanez A. M. Bustos R. I. (2016). Non-cytotoxic copper overload boosts mitochondrial energy metabolism to modulate cell proliferation and differentiation in the human erythroleukemic cell line K562. *Mitochondrion* 29 18–30. 10.1016/j.mito.2016.04.005 27094959

[B51] Samimi A. Khodayar M. J. Alidadi H. Khodadi E. (2020). The dual role of ROS in hematological malignancies: stem cell protection and cancer cell metastasis. *Stem Cell Rev. Rep.* 16 262–275. 10.1007/s12015-019-09949-5 31912368

[B52] Schell J. C. Rutter J. (2017). Mitochondria link metabolism and epigenetics in haematopoiesis. *Nat. Cell Biol.* 19 589–591. 10.1038/ncb3540 28561053

[B53] Schneider C. A. Rasband W. S. Eliceiri K. W. (2012). NIH Image to ImageJ: 25 years of image analysis. *Nat. Methods* 9 671–675. 10.1038/nmeth.2089 22930834 PMC5554542

[B54] Shaughnessy D. T. McAllister K. Worth L. Haugen A. C. Meyer J. N. Domann F. E. (2014). Mitochondria, energetics, epigenetics, and cellular responses to stress. *Environ. Health Perspect.* 122 1271–1278. 10.1289/ehp.1408418 25127496 PMC4256704

[B55] Son M. J. Kwon Y. Son M.-Y. Seol B. Choi H.-S. Ryu S.-W. (2015). Mitofusins deficiency elicits mitochondrial metabolic reprogramming to pluripotency. *Cell Death Differ.* 22 1957–1969. 10.1038/cdd.2015.43 25882047 PMC4816104

[B56] Tatapudy S. Aloisio F. Barber D. Nystul T. (2017). Cell fate decisions: emerging roles for metabolic signals and cell morphology. *EMBO Rep.* 18 2105–2118. 10.15252/embr.201744816 29158350 PMC5709733

[B57] Teslaa T. Teitell M. A. (2015). Pluripotent stem cell energy metabolism: an update. *EMBO J.* 34 138–153. 10.15252/embj.201490446 25476451 PMC4337063

[B58] Tian Y. Huang Z. Wang Z. Yin C. Zhou L. Zhang L. (2014). Identification of novel molecular markers for prognosis estimation of acute myeloid leukemia: over-expression of PDCD7, FIS1 and Ang2 May indicate poor prognosis in pretreatment patients with acute myeloid leukemia. *PLoS One* 9:e0084150. 10.1371/journal.pone.0084150 24416201 PMC3885535

[B59] ToddL. R.GomathinayagamR.SankarU. (2010). A novel Gfer-Drp1 link in preserving mitochondrial dynamics and function in pluripotent stem cells. *Autophagy* 6 821–822. 10.1091/mbc.E09-11-093720581476

[B60] Wai T. Langer T. (2016). Mitochondrial dynamics and metabolic regulation. *Trends Endocrinol. Metab.* 27 105–117. 10.1016/j.tem.2015.12.001 26754340

[B61] Wanet A. Arnould T. Najimi M. Renard P. (2015). Connecting mitochondria, metabolism, and stem cell fate. *Stem Cells Dev.* 24 1957–1971. 10.1089/scd.2015.0117 26134242 PMC4543487

[B62] Wang B. Nguyen M. Chang N. C. Shore G. C. (2011). Fis1, Bap31 and the kiss of death between mitochondria and endoplasmic reticulum. *EMBO J.* 30 451–452. 10.1038/emboj.2010.352 21285974 PMC3034021

[B63] Wang K. Long B. Jiao J.-Q. Wang J.-X. Liu J.-P. Li Q. (2012). miR-484 regulates mitochondrial network through targeting Fis1. *Nat. Commun.* 3:781. 10.1038/ncomms1770 22510686

[B64] Westermann B. (2010). Mitochondrial fusion and fission in cell life and death. *Nat. Rev. Mol. Cell Biol.* 11 872–884. 10.1038/nrm3013 21102612

[B65] Wilson T. J. Slupe A. M. Strack S. (2013). Cell signaling and mitochondrial dynamics: implications for neuronal function and neurodegenerative disease. *Neurobiol. Dis.* 51 13–26. 10.1016/j.nbd.2012.01.009 22297163 PMC3383441

[B66] Yamada S. Kubo Y. Yamazaki D. Sekino Y. Kanda Y. (2017). Chlorpyrifos inhibits neural induction via Mfn1-mediated mitochondrial dysfunction in human induced pluripotent stem cells. *Sci. Rep.* 7:40925. 10.1038/srep40925 28112198 PMC5256306

[B67] Yamada S. Yamazaki D. Kanda Y. (2018). 5-Fluorouracil inhibits neural differentiation via Mfn1/2 reduction in human induced pluripotent stem cells. *J. Toxicol. Sci.* 43 727–734. 10.2131/jts.43.727 30518710

[B68] Yamashita S.-I. Jin X. Furukawa K. Hamasaki M. Nezu A. Otera H. (2016). Mitochondrial division occurs concurrently with autophagosome formation but independently of Drp1 during mitophagy. *J. Cell Biol.* 215 649–665. 10.1083/jcb.201605093 27903607 PMC5147001

[B69] Youle R. J. van der Bliek A. M. (2012). Mitochondrial fission, fusion, and stress. *Science* 337 1062–1065. 10.1126/science.1219855 22936770 PMC4762028

[B70] Yu R. Jin S. B. Lendahl U. Nistér M. Zhao J. (2019). Human Fis1 regulates mitochondrial dynamics through inhibition of the fusion machinery. *EMBO J.* 38 1421–1437. 10.15252/embj.201899748 30842096 PMC6463211

[B71] Zhang J. (2003). Role of Ras signaling in erythroid differentiation of mouse fetal liver cells: functional analysis by a flow cytometry-based novel culture system. *Blood* 102 3938–3946. 10.1182/blood-2003-05-1479 12907435

[B72] Zhang J. Khvorostov I. Hong J. S. Oktay Y. Vergnes L. Nuebel E. (2011). UCP2 regulates energy metabolism and differentiation potential of human pluripotent stem cells. *EMBO J.* 30 4860–4873. 10.1038/emboj.2011.401 22085932 PMC3243621

