# Supplementary figures and images for: Erythroid Differentiation and Heme Biosynthesis Are Dependent on a Shift in the Balance of Mitochondrial Fusion and Fission Dynamics
Source: Front Cell Dev Biol. 2020 Nov 23;8:592035. doi: 10.3389/fcell.2020.592035 (PMC7719720; doi:10.3389/fcell.2020.592035)

Supplementary Figure 1

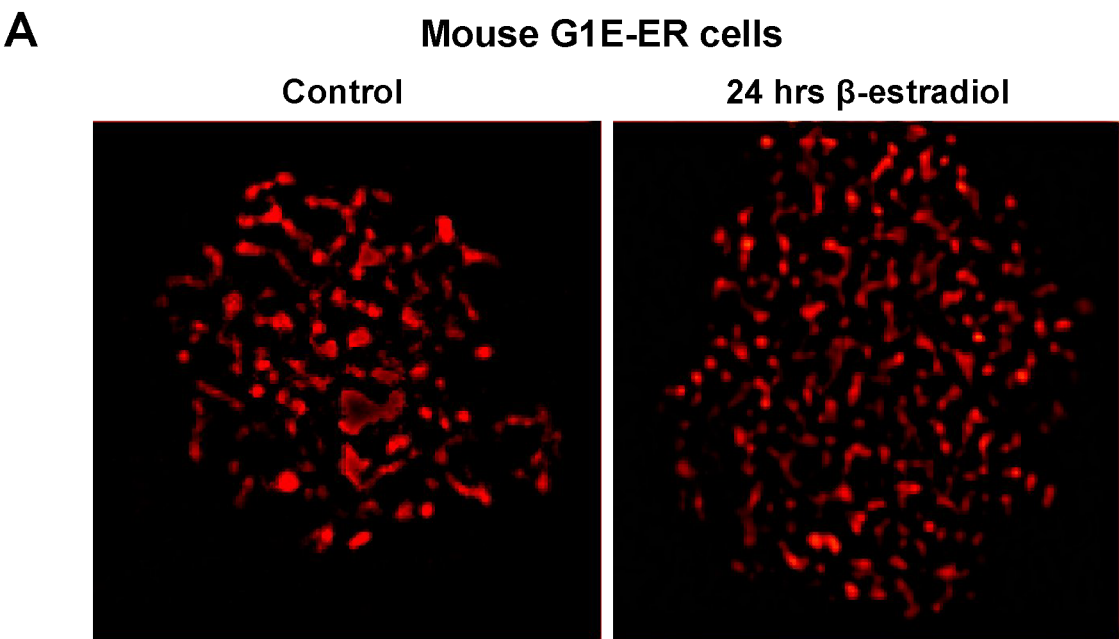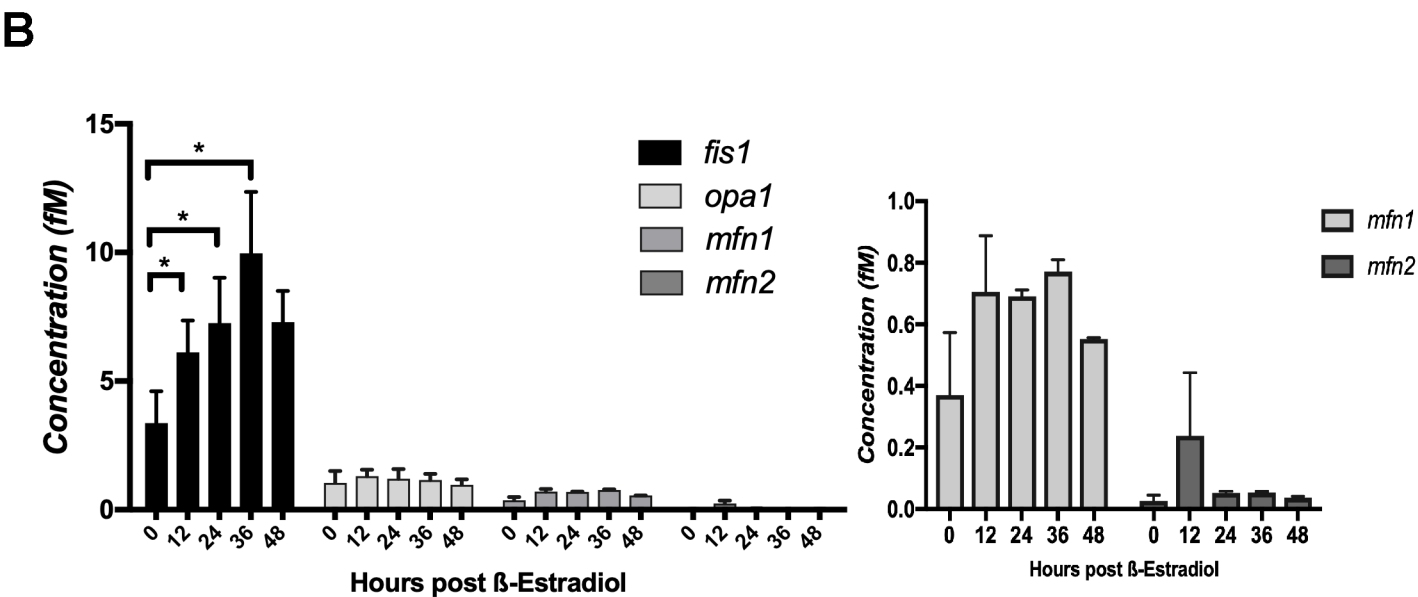

Supplement: Supplementary Figure 1 — Mitochondrial dynamics in mouse erythropoiesis. (A) Visualization of mitochondrial morphology. The mouse erythropoietic cell line G1E-ER were β-estradiol-treated to induced erythroid differentiation. Mitochondria were stained with 10 nM MitoTracker Red CMXRos and visualized by confocal microscopy at 24 h post induction. It is shown one cell per condition. The mitochondrial web increased its biomass and became fragmented upon erythroid differentiation. (B) Mitochondrial Dynamics gene expression. mRNA expression kinetics of mitochondrial dynamics genes Fis1, Opa1, Mnf1, Mfn2 during β-estradiol-induced erythropoiesis in G1E-ER cells, was assessed by quantitative real time (qRT-PCR). Cells were collected at 0, 12, 24, 36, and 48 h. The left panel shows the all four genes and the right panel, just Mfn1 and Mfn2 genes at higher scale. Average expression values are ±STD of n = 3. Statistical analysis was ANOVA followed by Bonferroni post hoc test (p < 0.05). [file Data_Sheet_1.PDF]

Supplementary 2

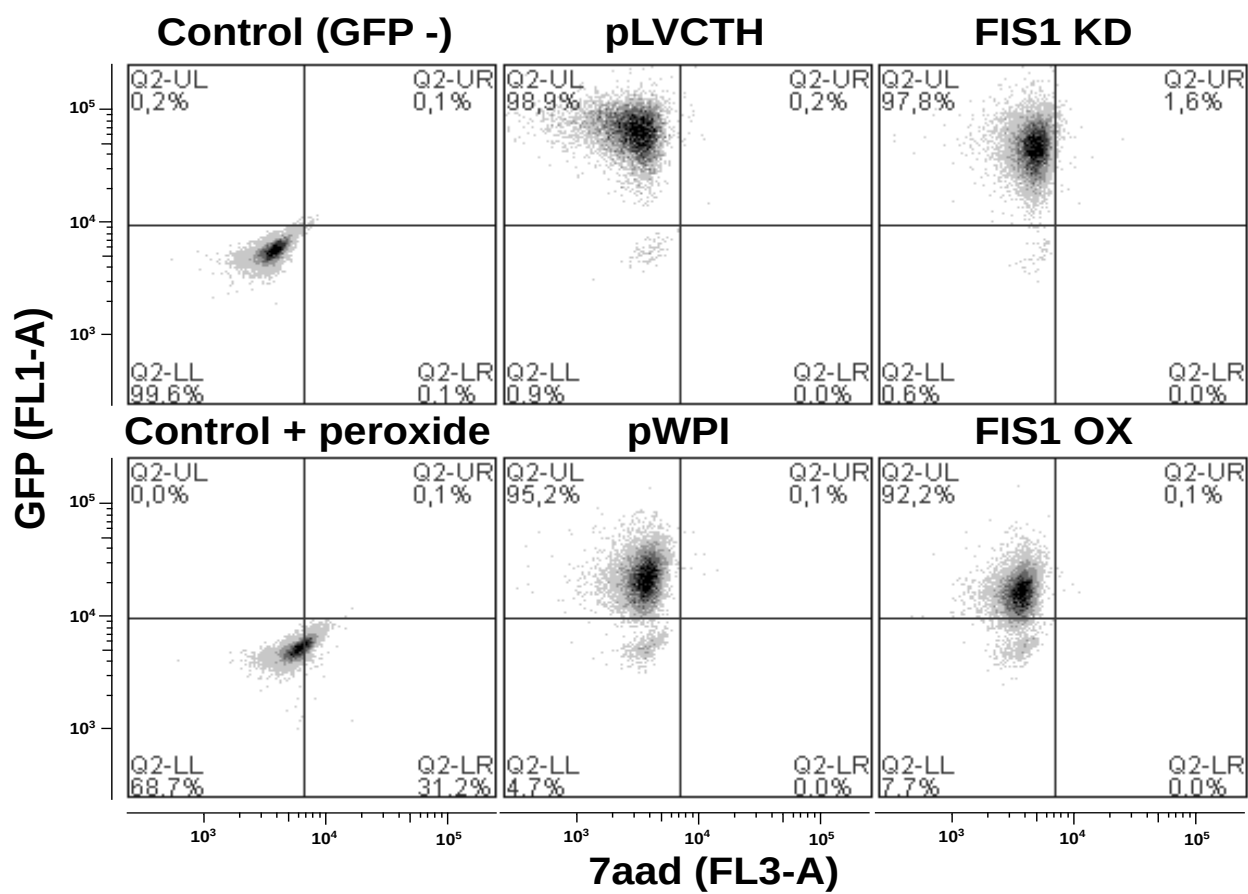

Supplement: Supplementary Figure 2 — Lentiviral transduction of CD34+ cells. CD34+ cells were lentivirus-transduced one hour after isolation overnight. 3 days later, transduction efficiency was assessed by flow cytometry. GFP expression was used as indicator of successful transduction. In addition, cells were stained with 7-AAD to assess dead cells. Hydrogen Peroxide was used in control cells as a positive control of dead cells. Transduction efficiency of CD34+ was over 90% with no dead cells (<1%). [file Data_Sheet_2.PDF]

Supplementary 3

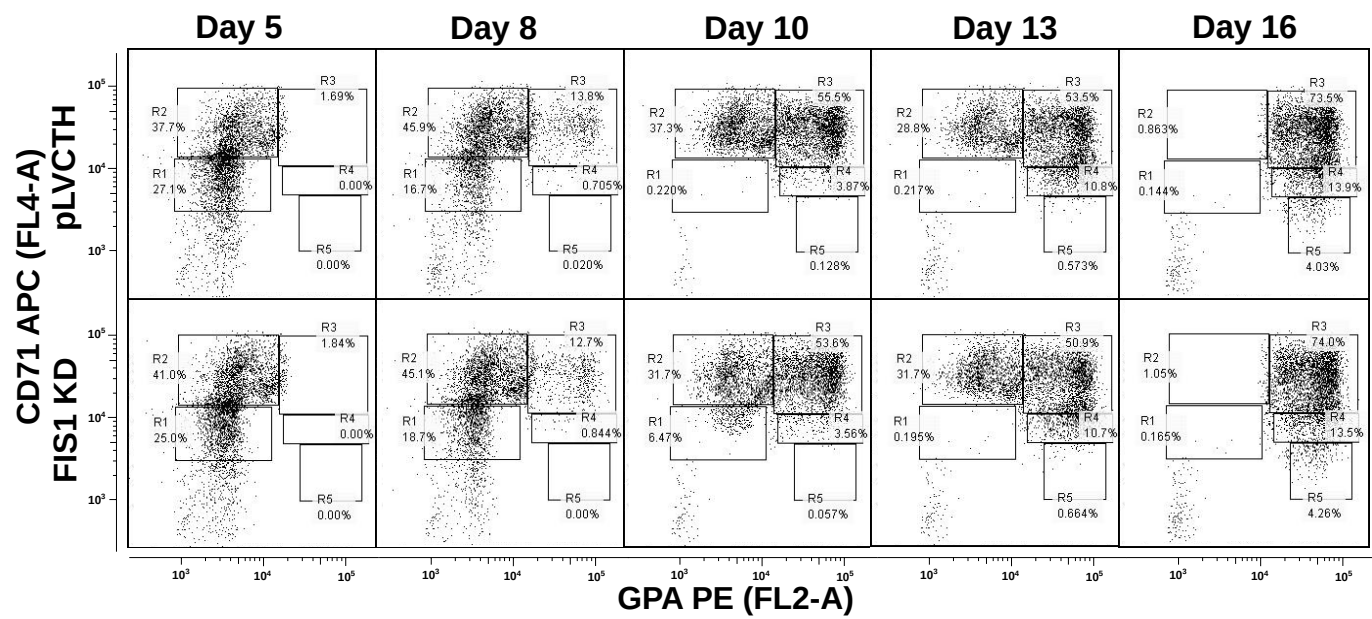

Supplement: Supplementary Figure 3 — Erythroid Progression in FIS1 KD cells. Flow cytometry analysis of surface markers anti-CD71-APC and anti-GPA-PE in pLVCTH control and FIS1 KD cells at D5, D8, D10, D12, and D16 of EPO-induced erythroid differentiation (n = 3). [file Data_Sheet_3.PDF]

Supplementary 4

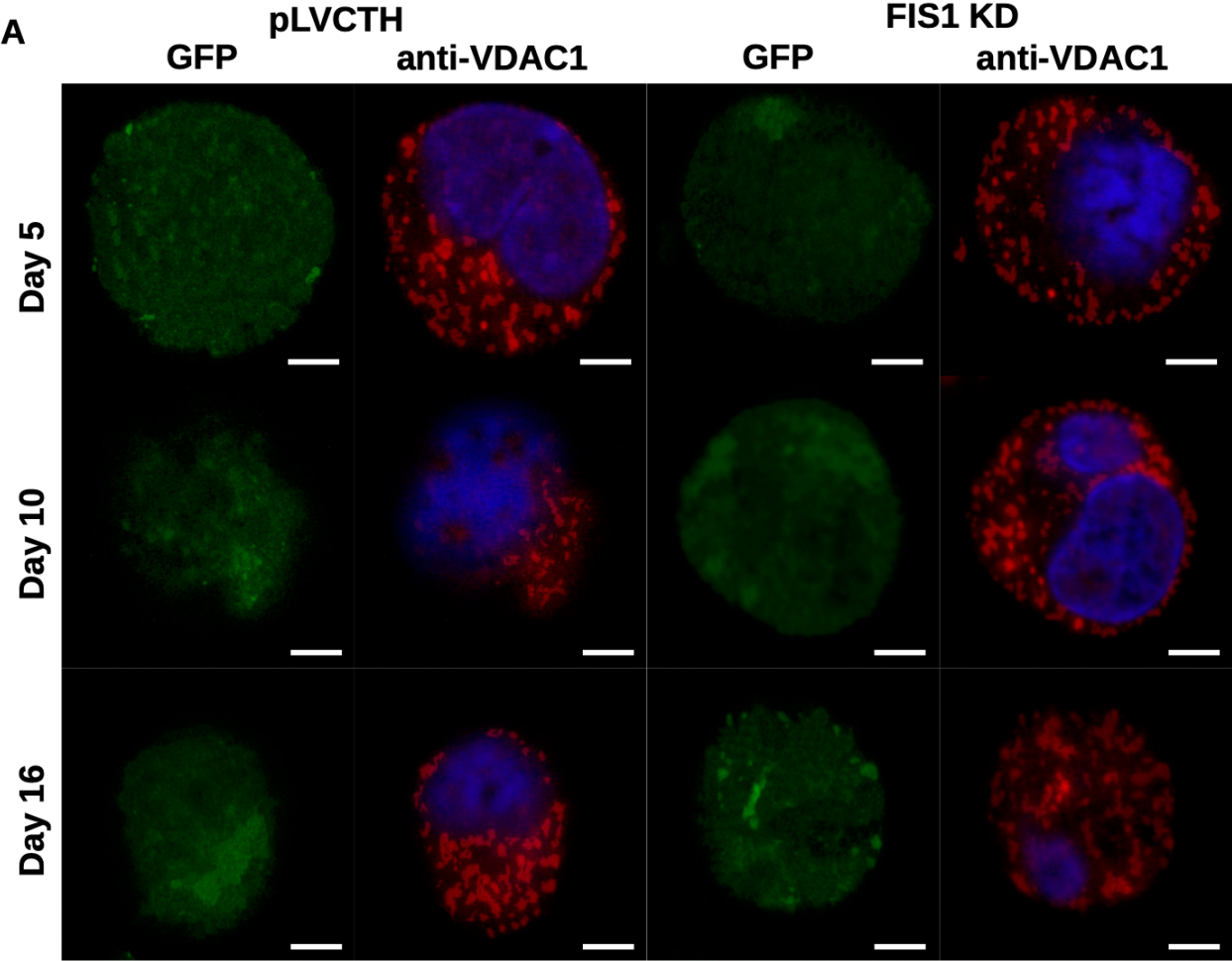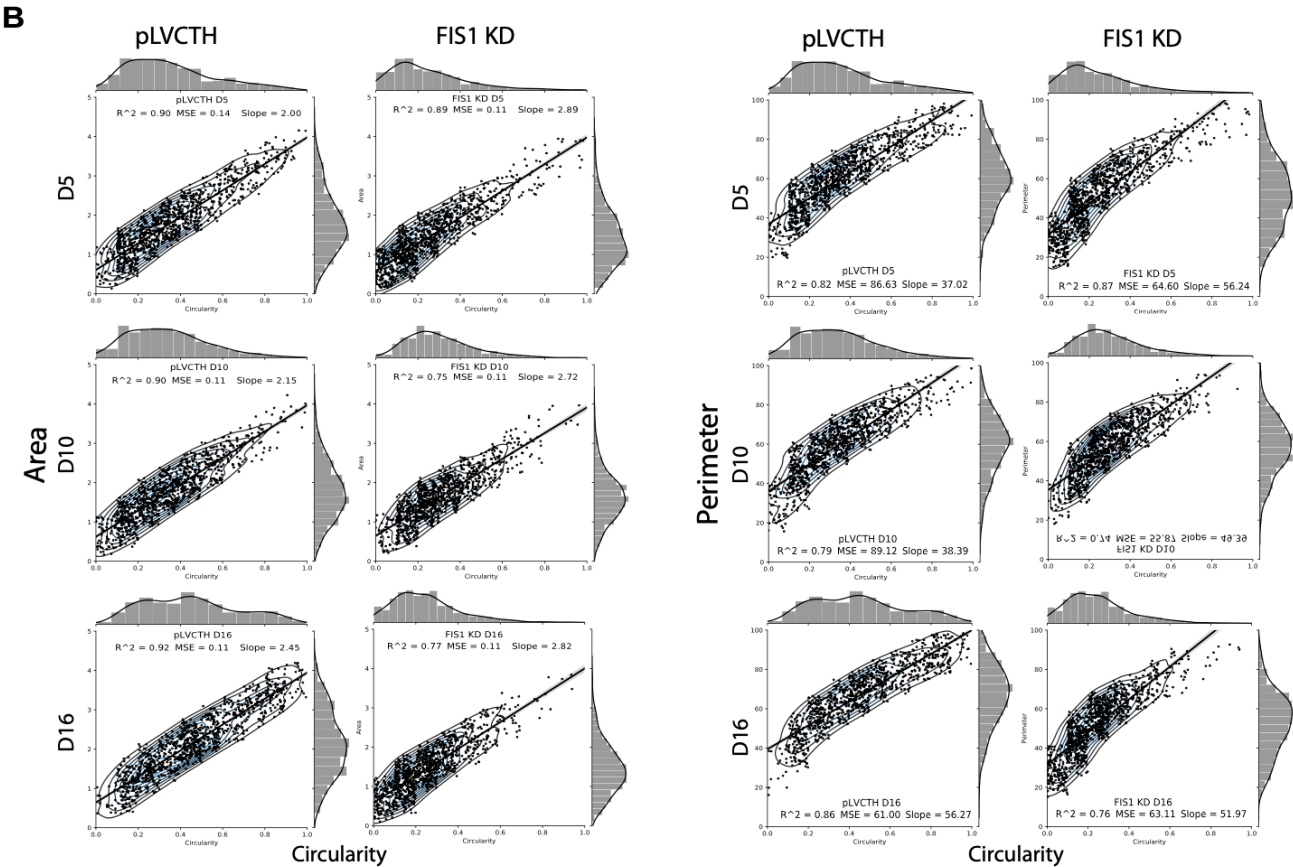

Supplement: Supplementary Figure 4 — Effects of FIS1 KD on mitochondrial morphology. (A) Mitochondria visualization by confocal microscopy. Immunofluorescence microscopy at D5, D10, and D16 of erythroid differentiation for pLVCTH control and FIS1 KD cells. Anti-VDAC1 antibody labeled mitochondria (red) and the DAPI dye, the nucleus (blue). (B) Mitochondrial morphometric analysis in term of Area at D5, D10, and D16 of differentiation for control pWPI and FIS1 KD cells. It was performed using the Z-slides from GFP+ cells. Each dot represents a mitochondrial unit in terms of Area (Y axes) and Circularity (X axes). Also, a frequency histogram of mitochondrial circularity and area were added. Linear regression analysis was performed to compare slopes, which were significantly different (p < 0.05). R2 and MSE were also calculated. [file Data_Sheet_4.PDF]
